# Club cells form lung adenocarcinomas and maintain the alveoli of adult mice

**DOI:** 10.7554/eLife.45571

**Published:** 2019-05-29

**Authors:** Magda Spella, Ioannis Lilis, Mario AA Pepe, Yuanyuan Chen, Maria Armaka, Anne-Sophie Lamort, Dimitra E Zazara, Fani Roumelioti, Malamati Vreka, Nikolaos I Kanellakis, Darcy E Wagner, Anastasios D Giannou, Vasileios Armenis, Kristina AM Arendt, Laura V Klotz, Dimitrios Toumpanakis, Vassiliki Karavana, Spyros G Zakynthinos, Ioanna Giopanou, Antonia Marazioti, Vassilis Aidinis, Rocio Sotillo, Georgios T Stathopoulos

**Affiliations:** 1 Laboratory for Molecular Respiratory Carcinogenesis, Department of Physiology, Faculty of MedicineUniversity of PatrasRioGreece; 2 Comprehensive Pneumology Center (CPC), Institute for Lung Biology and Disease (iLBD)University Hospital, Ludwig-Maximilians University, Helmholtz Center Munich, The German Center for Lung Research (DZL)MunichGermany; 3 Division of Molecular Thoracic Oncology, Translational Lung Research Center (TLRC)German Cancer Research Center (DKFZ), The German Center for Lung Research (DZL)HeidelbergGermany; 4 Institute of ImmunologyBiomedical Sciences Research Center "Alexander Fleming"VariGreece; 5 First Department of Critical Care Medicine and Pulmonary Services, School of MedicineEvangelismos Hospital, National and Kapodistrian University of AthensAthensGreece; King's College LondonUnited Kingdom; Calico Life SciencesUnited States

**Keywords:** lung adenocarcinoma, chemical carcinogenesis, urethane, airway transcriptome, Human, Mouse

## Abstract

Lung cancer and chronic lung diseases impose major disease burdens worldwide and are caused by inhaled noxious agents including tobacco smoke. The cellular origins of environmental-induced lung tumors and of the dysfunctional airway and alveolar epithelial turnover observed with chronic lung diseases are unknown. To address this, we combined mouse models of genetic labeling and ablation of airway (club) and alveolar cells with exposure to environmental noxious and carcinogenic agents. Club cells are shown to survive *KRAS* mutations and to form lung tumors after tobacco carcinogen exposure. Increasing numbers of club cells are found in the alveoli with aging and after lung injury, but go undetected since they express alveolar proteins. Ablation of club cells prevents chemical lung tumors and causes alveolar destruction in adult mice. Hence club cells are important in alveolar maintenance and carcinogenesis and may be a therapeutic target against premalignancy and chronic lung disease.

## eLife digest

The deadliest form of lung cancer is called lung adenocarcinoma, or LUAD. Tobacco chemicals often cause the disease by damaging the genetic information of lung cells. The damage leads to harmful changes in the DNA sequence which prompt the cells to form tumors. For instance, the most common of these changes takes place in a gene called *KRAS*. However, it is still unclear exactly which type of lung cells are more likely to develop into a tumor.

In the lungs, airway epithelial cells cover the inside of the passages that bring the air inside little sacks called alveoli, which are lined by alveolar cells. Previous studies have used genetic methods to switch on the *KRAS* mutation in different compartments of the mouse lung. This showed that groups of airway cells, of alveolar cells, and of a class of cells located at the junction between airways and alveoli could all give rise to cancer. However, these experiments did not examine how tobacco chemicals could give rise to tumors in different groups of lung cells.

Here, Spella et al. triggered LUAD in adult mice by exposing them to the toxic chemicals found in tobacco smoke, but without making any change to the *KRAS* gene. These mice also had genetically engineered reporters that could be used to deduce where the resulting tumors came from. DNA sequencing showed that the airway epithelial cells gained *KRAS* mutations after the chemical treatment. When the airway epithelial cells were experimentally removed before the treatments with tobacco chemicals, these mice did not get LUAD tumors. Spella et al. also observed that the tobacco-induced tumors came from the epithelial cells in the airways, and not from the cells in the alveoli. Moreover, when the lung was damaged, airway cells could move to the alveoli and start adopting the identity of alveolar cells, thereby replenishing this population. Together, these experiments imply that tobacco-induced LUAD starts in the airway epithelial cells.

These findings suggest that airway epithelial cells could be targeted to stop lung cancer early on. Further studies should also examine how airway epithelial cells can transition to look more like alveolar cells when the lungs get harmed.

## Introduction

Chronic lung diseases present tremendous health burdens attributed to dysfunctional alveolar repair (Barnes et al., 2015; Lozano et al., 2012; Spella et al., 2017). Lung adenocarcinoma (LUAD), the leading cancer killer worldwide, is mainly caused by chemical carcinogens of tobacco smoke that induce mutations of the Kirsten rat sarcoma viral oncogene homologue (*KRAS*) in yet unidentified pulmonary cells (Torre et al., 2015; Forbes et al., 2011; Hecht, 1999; Campbell et al., 2016; Cancer Genome Atlas Research Network, 2014). The discovery of the cellular lineages and the transcriptional programs that underlie lung regeneration and carcinogenesis is extremely important, since epithelial developmental pathways are intimately related with oncogenic signaling to jointly regulate stemness and drug resistance (Barbie et al., 2009; Seguin et al., 2014). To this end, lineage-specific genes encoding epithelial proteins that support the physiological functions of the lungs were recently shown to suffer non-coding insertions and deletions in LUAD, lending further support to the longstanding notion that epithelial cells that express lung-restricted proteins are the cellular sources of LUAD (Imielinski et al., 2017).

**Figure 1.**
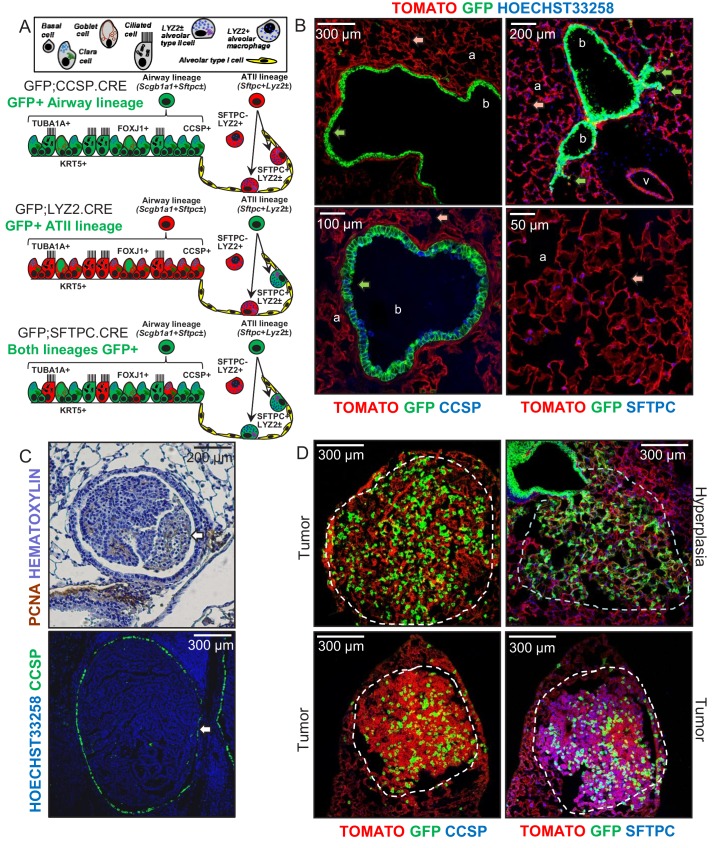
Airway cells in urethane-induced lung tumors. (**A**) Cartoon of the different lung epithelial lineages, their distribution in the airways (club, goblet, ciliated, and basal cells) and the alveoli (alveolar type I and II cells), their permanent fluorescent genetic labeling in the reporter mice used in this study (green color), and the protein markers used for their identification. See also Figure 1—figure supplements 1–5. (**B**) Lung sections from naïve 6-week-old GFP;CCSP.CRE mice (*n* = 22), in which all airway cells bear permanent genetic GFP+ (green arrows) and all other cells TOMATO+ (red arrows) labels, counterstained with nuclear Hoechst33258 dye (top) or immunostained for the club cell marker CCSP and the alveolar type II cell marker SFTPC (bottom). a, alveoli; b, bronchi; v, vein. See also Figure 1—figure supplements 6–8. (**C**) Proliferating cell nuclear antigen (PCNA; brown) and hematoxylin (blue)-stained (top) and CCSP (green) and Hoechst33258 (blue)-stained (bottom) lung tumor sections of urethane-treated C57BL/6 mice six months post-treatment (*n* = 5/group), depicting endobronchial lung adenocarcinomas (white arrows). See also Figure 1—figure supplements 9–11. (**D**) Lung sections of GFP;CCSP.CRE mice (*n* = 10) at six months post-urethane treatment bearing hyperplasias and tumors (dashed outlines, top), and immunostained for the club cell marker CCSP (bottom left) and the alveolar type II cell marker SFTPC (bottom right). Note the GFP-labeled lesions of airway origin that have lost CCSP and have acquired SFTPC immunoreactivity. See also Figure 1—figure supplements 12–19. CCSP, Clara cell secretory protein; TUBA1A, acetylated α-tubulin; SFTPC, surfactant protein C; LYZ2, lysozyme 2; FOXJ1, forkhead box J1; KRT5, keratin 5.

However, these cells of origin of LUAD remain only partially charted. Previous pulmonary lineage tracing studies that utilized noxious insults and ectopic expression of oncogenes in the respiratory epithelium incriminated both airway and alveolar cells as progenitors of newly formed alveoli and/or LUAD in adult mice (Zuo et al., 2015; Kim et al., 2005; Cho et al., 2011; Xu et al., 2012; Sutherland et al., 2014; Mainardi et al., 2014; Desai et al., 2014). To this end, airway epithelial cells (AEC) line the bronchi and include ciliated, basal, goblet, and Clara or club cells; alveolar type II cells (ATII) and alveolar macrophages (AMΦ) are distributed across the distal lung parenchyma; and bronchoalveolar stem cells (BASC) with dual AEC/ATII properties are located at the bronchoalveolar junctions. Established markers currently used to label these pulmonary lineages include acetylated tubulin (TUBA1A) for ciliated cells, keratin 5 (KRT5) for basal cells, forkhead box J1 (FOXJ1) for goblet cells, Clara cell secretory protein (CCSP) for club cells, surfactant protein C (SFTPC) and lysozyme 2 (LYZ2) for ATII cells, and LYZ2 for AMΦ, are summarized in Figure 1A and Figure 1—figure supplement 1, and are extensively studied in Desai et al. (2014) and Treutlein et al. (2014). However, existing mouse models for lineage tracing feature incomplete and/or promiscuous lung cell labeling, that is cellular markings fail to identify all cells of a target lineage (false negative marking) or wrongfully identify other cells outside of the target lineage (false positive marking) (Zuo et al., 2015; Kim et al., 2005; Cho et al., 2011; Xu et al., 2012; Sutherland et al., 2014; Mainardi et al., 2014; Desai et al., 2014). In addition, all studies that attempted to address the cellular origins of LUAD to date employed overexpression of oncogenes such as *KRAS*^G12D^ in the lungs, to conclude that ATII cells or BASC are the most probable culprits of the disease (Kim et al., 2005; Cho et al., 2011; Xu et al., 2012; Sutherland et al., 2014; Mainardi et al., 2014; Desai et al., 2014). However, it was recently shown that oncogenic *KRAS*^G12D^-driven mouse lung tumors do not imitate the mutational landscape of human LUAD as closely as tobacco carcinogen-induced LUAD do (Campbell et al., 2016; Cancer Genome Atlas Research Network, 2014; Westcott et al., 2015).

**Figure 1—figure supplement 1.**
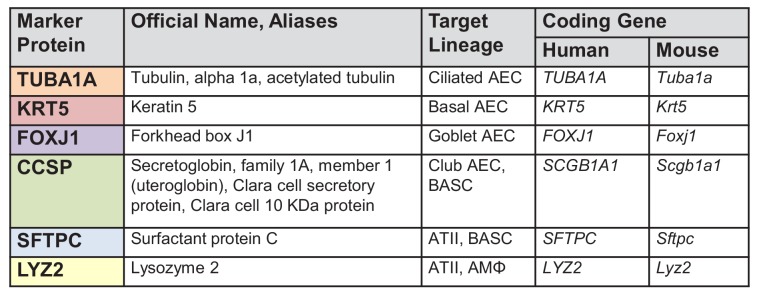
Table of pulmonary lineage markers and key abbreviations used in this study. TUBA1A, Tubulin alpha 1a or acetylated tubulin; KRT5, Keratin 5; FOXJ1, Forkhead box J1; CCSP, Secretoglobin, family 1A, member 1 (uteroglobin) or Clara cell secretory protein or Clara cell 10 KDa protein; SFTPC, Surfactant protein C; LYZ2, Lysozyme 2; AEC, airway epithelial cells; BASC, bronchoalveolar stem cells; ATII, alveolar type II cells or type II pneumocytes; AMΦ, alveolar macrophages.

**Figure 1—figure supplement 2.**
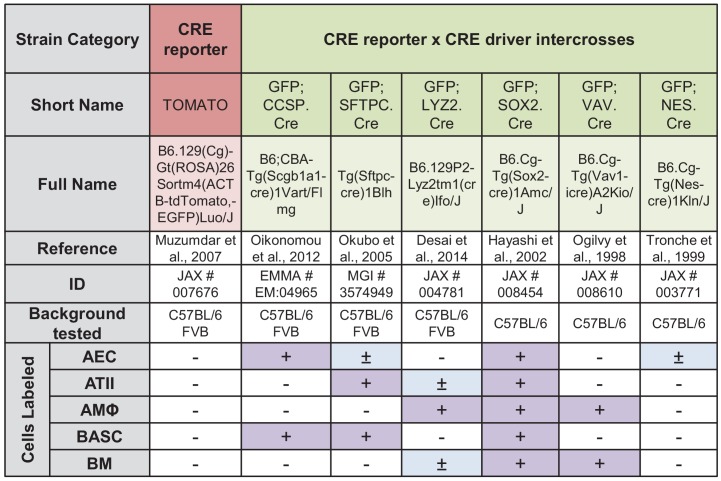
Genetic labeling of pulmonary lineages in eleven mouse strains and intercrosses: summary of results. CRE, causes recombination; TOMATO, tdTomato; GFP, green fluorescent protein; CCSP, Clara cell secretory protein; SFTPC, surfactant protein C; LYZ2, lysozyme 2; SOX2, sex determining region Y (SRY)-box 2; VAV, Vav Guanine Nucleotide Exchange Factor 1; NES, nestin; JAX, Jackson Laboratories; EMMA, European Mutant Mouse Archive; MGI, Mouse Genome Informatics; AEC, airway epithelial cells; BASC, bronchoalveolar stem cells; ATII, alveolar type II cells or type II pneumocytes; AMΦ, alveolar macrophages; ΒM, bone marrow (myeloid) cells. Symbols indicate: - (white), no genetic labeling; + (magenta), complete genetic labeling; ± (blue), partial genetic labeling.

**Figure 1—figure supplement 3.**
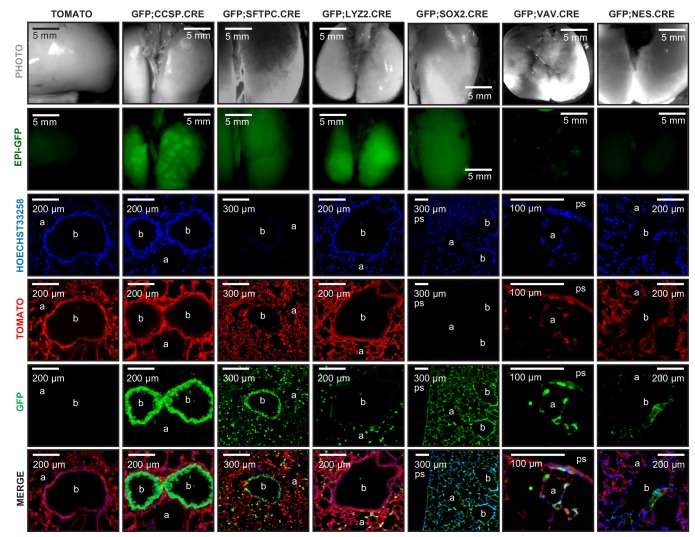
Genetic labeling of pulmonary lineages in seven lineage reporter strains on the C57BL/6 background: representative images. Representative photographs (top row) and green epifluorescence images (second row) of whole lungs, as well as fluorescent microscopic images of lung sections for nuclear Hoechst33258 stain (third row), endogenous TOMATO (fourth row), endogenous GFP (fifth row), and merged images (bottom row) of genetically marked mice on the C57BL/6 background employed in these studies (described in detail in Figure 1—figure supplement 2) at six postnatal weeks (*n* = 5/mouse strain). b, bronchi; a, alveoli; ps, pleural space.

**Figure 1—figure supplement 4.**
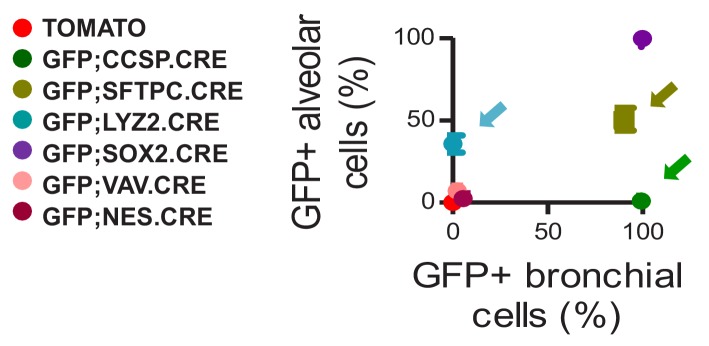
Genetic labeling of pulmonary lineages in seven lineage reporter strains on the C57BL/6 background: data summary. XY plot of GFP-labeled airway versus alveolar cells from *n* = 5 mice/mouse strain. Arrows denote the three lineage-reporter strains selected for further study including GFP;CCSP.CRE (green), GFP;LYZ2.CRE (blue), and GFP;SFTPC.CRE (olive) mice. Data are given as mean ± SD. 10.7554/eLife.45571.008Figure 1—figure supplement 4—source data 1.Quantification of GFP+ alveolar and bronchial cells in our reporter mice.

**Figure 1—figure supplement 5.**
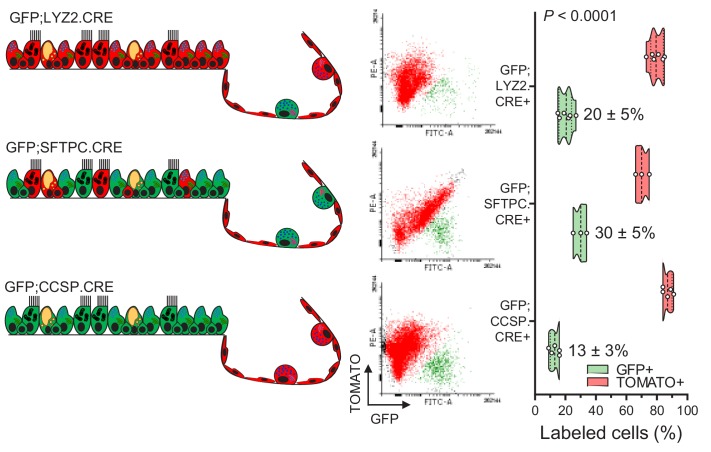
Flow cytometric quantification of lineage-labeled cells in three lineage reporter strains on the C57BL/6 background. Schematic representation of genetic lineage labeling of GFP;CCSP.CRE, GFP;SFTPC.CRE, and GFP;LYZ2.CRE mice (left), flow cytometric gating strategy to quantify GFP+ and TOMATO+ cells (middle), and violin plot from *n* = 5, 3, and six mice/strain (right). Numbers are mean ± SD. *P*, overall probability, two-way ANOVA. 10.7554/eLife.45571.010Figure 1—figure supplement 5—source data 1.Flow cytometric quantification of GFP+ and TOMATO+ cells in three lineage reporter mice.

**Figure 1—figure supplement 6.**
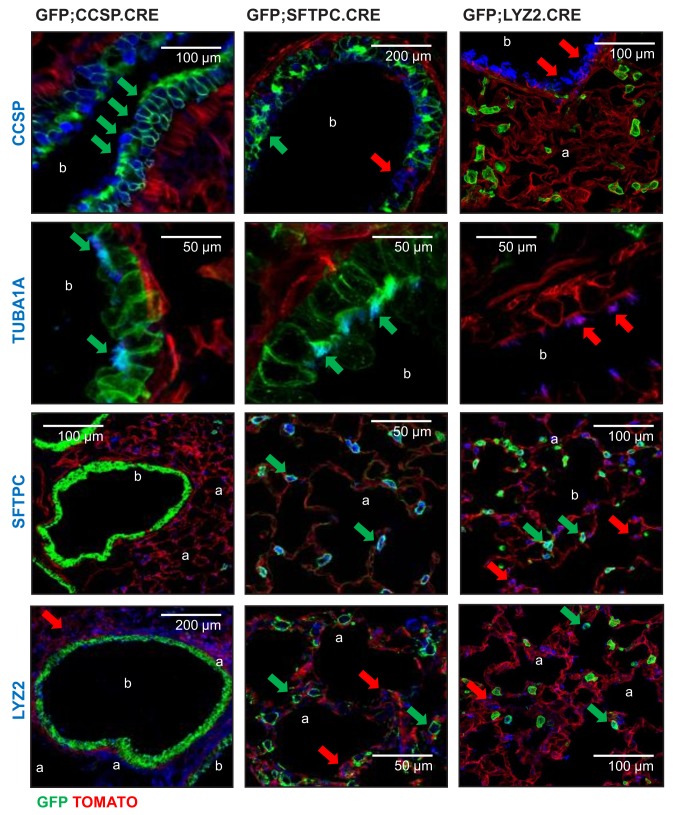
Genetic lineage labels of protein-marked cells in three lineage reporter strains on the C57BL/6 background: representative images. Representative merged fluorescent microscopic images from lineage marker-stained lung sections of 6-week-old lineage-labeled mice (*n* = 5/group). Arrows indicate cells expressing the respective marker protein with (green) or without (red) genetic lineage-labeling. CCSP, Clara cell secretory protein; TUBA1A, acetylated tubulin; SFTPC, surfactant protein C; LYZ2, lysozyme 2; b, bronchi; a, alveoli.

**Figure 1—figure supplement 7.**
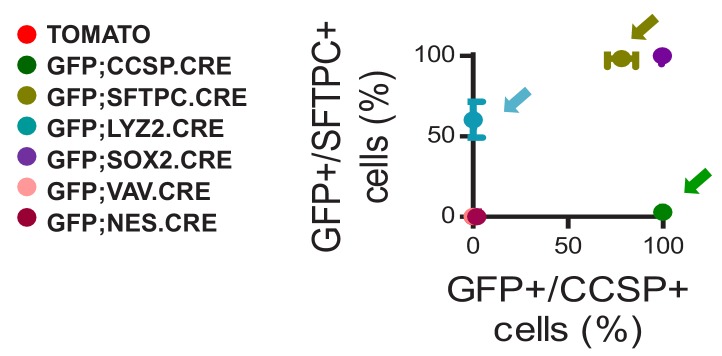
Genetic lineage labels of protein-marked cells in seven lineage reporter strains on the C57BL/6 background: data summary. XY plot of ratios of genetic GFP-labeled to protein marker CCSP and SFTPC-immunoreactive cells (*n* = 5/group). Arrows denote the three lineage-reporter strains selected for further study including GFP;CCSP.CRE (green), GFP;LYZ2.CRE (blue), and GFP;SFTPC.CRE (olive) mice. Data are given as mean ± SD. 10.7554/eLife.45571.013Figure 1—figure supplement 7—source data 1.Quantification of GFP+/SFTPC+ and GFP+/CCSP+ cells in our reporter mice.

**Figure 1—figure supplement 8.**
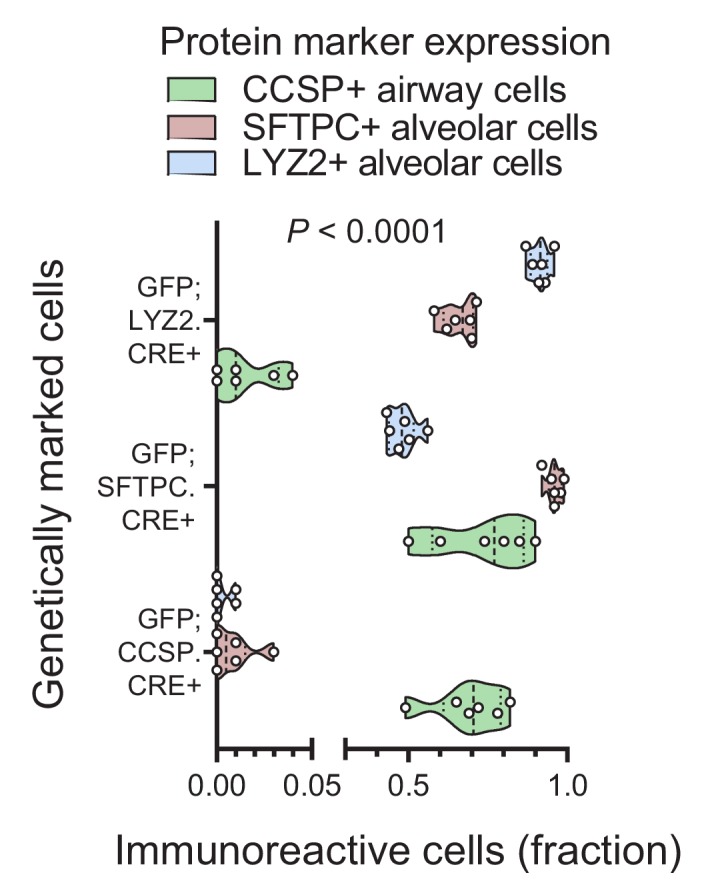
Protein markings of lineage-labeled cells in three lineage reporter strains on the C57BL/6 background: data summary. Quantification of protein marker expression of genetic-labeled cells of GFP;CCSP.CRE, GFP;LYZ2.CRE, and GFP;SFTPC.CRE mice (*n* = 6/strain) for Clara cell secretory protein (CCSP), surfactant protein C (SFTPC), and lysozyme 2 (LYZ2). Data are given as violin plots. *P*, overall probability, two-way ANOVA. 10.7554/eLife.45571.015Figure 1—figure supplement 8—source data 1.Quantification of protein marker expression of GFP+ cells in three lineage reporter mice.

**Figure 1—figure supplement 9.**
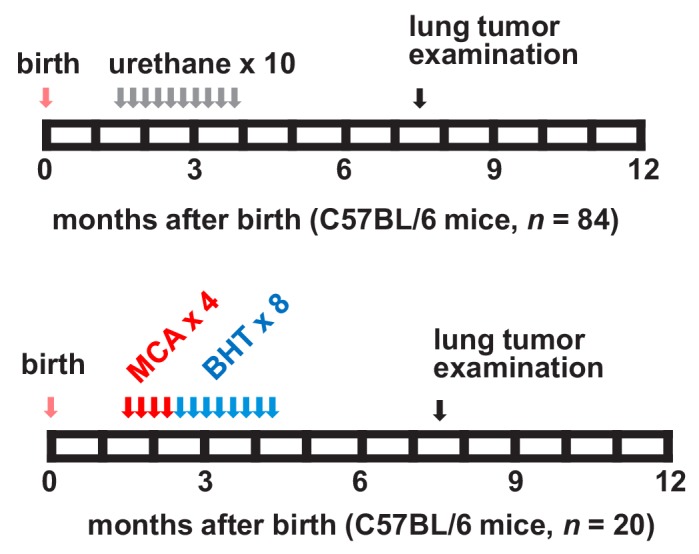
Two carcinogen regimens for reproducible lung tumor induction in naturally resistant C57BL/6 mice. Top: schematic of multi-hit urethane administration tailored to yield 90% tumor incidence in C57BL/6 mice: ten weekly intraperitoneal injections of 1 g/Kg urethane (ethyl carbamate, EC; gray arrows) are initiated at six weeks after birth (pink arrow) and lungs are examined six months after the first urethane injection (black arrow). Bottom: 3-methyl-1,2-dyhydrobenzo[j]aceanthrylene (MCA)/butylated hydroxytoluene (BHT) regimen tailored to yield 90% tumor incidence in C57BL/6 mice. Four weekly intraperitoneal injections of 15 mg/Kg MCA (red arrows) initiated at six weeks after birth (pink arrow) are followed by eight weekly intraperitoneal injections of 200 mg/Kg BHT (blue arrows) and lung examination at six months after first MCA dose (black arrow).

**Figure 1—figure supplement 10.**
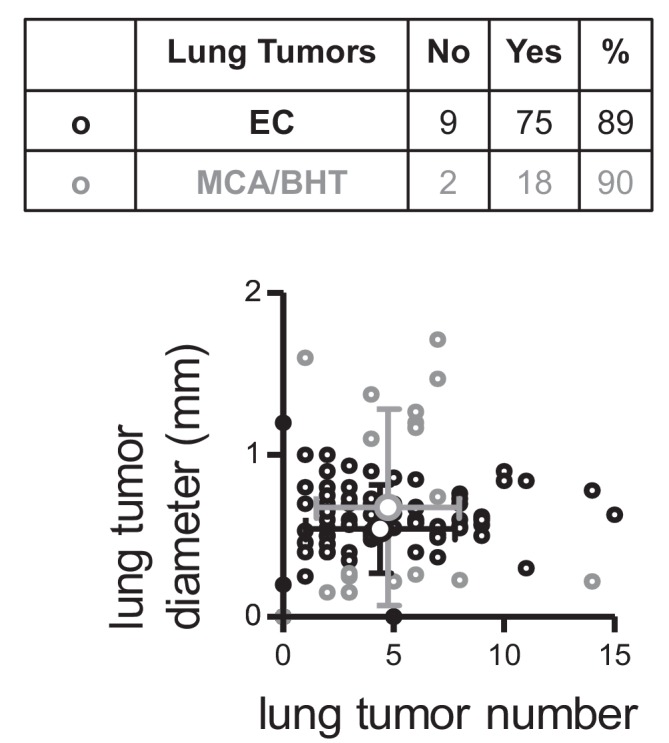
Lung tumors induced in C57BL/6 mice by two carcinogen regimens. Eighty-four C57BL/6 mice received ten weekly intraperitoneal injections of 1 g/Kg urethane (ethyl carbamate, EC) initiated at six weeks of age and lungs were examined six months after the first urethane injection (black font and symbols). Twenty C57BL/6 mice received four weekly intraperitoneal injections of 15 mg/Kg 3-methyl-1,2-dyhydrobenzo[j]aceanthrylene (MCA) followed by eight weekly intraperitoneal injections of 200 mg/Kg butylated hydroxytoluene (BHT) and lungs were examined six months after the first MCA dose (gray font and symbols). Table shows tumor incidence and graph shows tumor number versus mean tumor diameter. Each small circle represents one mouse and each large circle with error bar the means for each carcinogen regimen. 10.7554/eLife.45571.018Figure 1—figure supplement 10—source data 1.Quantification of data shown in Figure 1—figure supplement 10.

**Figure 1—figure supplement 11.**
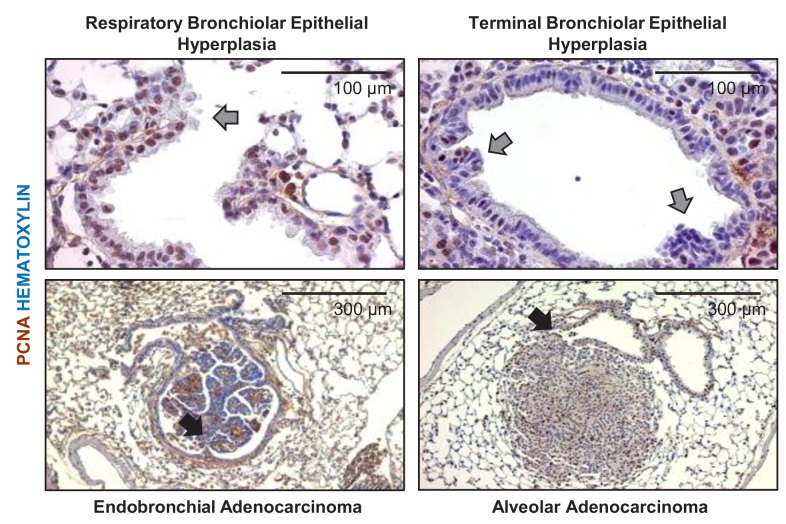
Airway links of urethane-induced lung adenocarcinomas. Proliferating cell nuclear antigen (PCNA)-stained lung sections of urethane-treated C57BL/6 mice at six months post-treatment start. Arrows: airway hyperplasias (gray) and lung adenocarcinomas (black) arising within a bronchus (left) and apparently in an alveolar region but adjacent to a bronchus (right).

**Figure 1—figure supplement 12.**
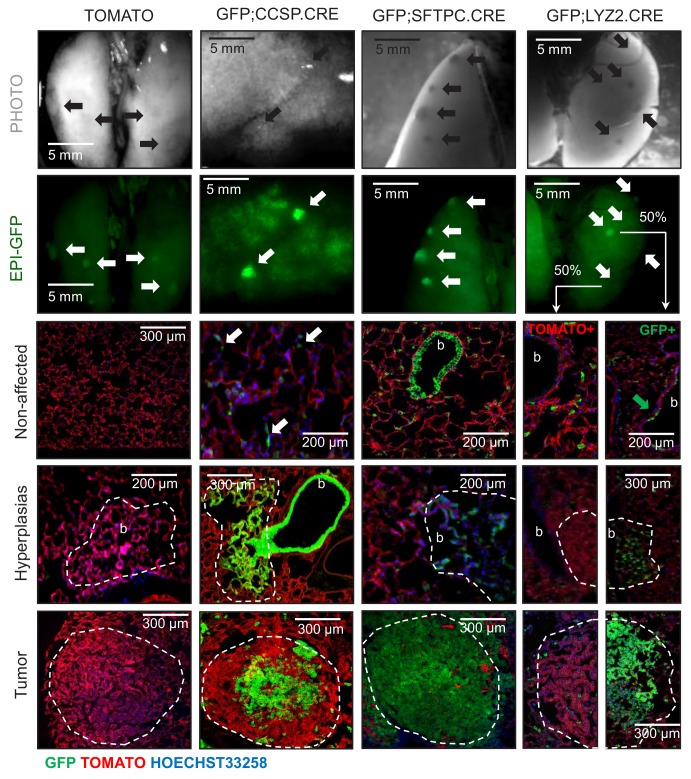
Genetic labeling of urethane-induced lung adenocarcinomas in four lineage reporter strains on the C57BL/6 background: representative images. Representative photographs (top row) and green epifluorescence images (second row), as well as merged fluorescent microscopic images of lung sections for nuclear Hoechst33258 stain, endogenous TOMATO, and endogenous GFP (bottom three rows), of tumor-bearing lungs from genetically marked mice employed in these studies (described in detail in Figure 1—figure supplement 2) at six months after initiation of ten weekly intraperitoneal urethane injections (*n* = 30, 22, 18, and 20/strain, respectively). b, bronchi. Top two rows: arrows indicate lung tumors. Bottom three rows: white arrows indicate GFP-labeled cells in apparently non-affected alveolar areas of GFP;CCSP.CRE mice; green arrow indicates rare GFP+ cell in non-affected central airway of GFP;LYZ2.CRE mouse. Note the absence of GFP-labeling of lung tumors in TOMATO mice, the complete GFP-labeling in GFP;CCSP.CRE and GFP;SFTPC.CRE mice, and the partial GFP-labeling in GFP;LYZ2.CRE mice.

**Figure 1—figure supplement 13.**
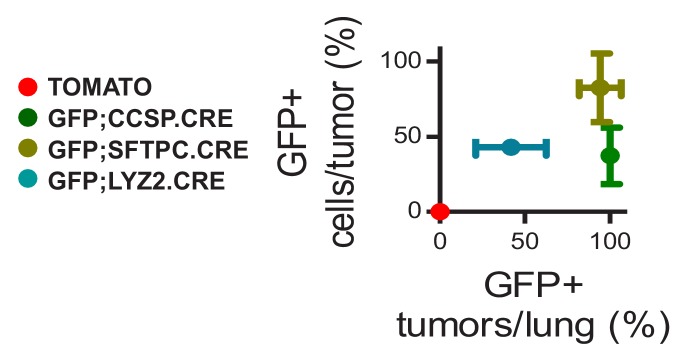
Genetic labeling of urethane-induced lung adenocarcinomas in four lineage reporter strains on the C57BL/6 background: data summary. XY plot of percentage of GFP-labeled tumors/lung versus GFP-labeled tumor cells/tumor averaged per lung in strains from Figure 1—figure supplement 12 (*n* = 30, 22, 18, and 20/group, respectively). Data are given as mean ± SD. 10.7554/eLife.45571.022Figure 1—figure supplement 13—source data 1.Quantification of GFP+ tumors/lung and GFP+ cells/tumor in four lineage reporter mice after urethane exposure.

**Figure 1—figure supplement 14.**
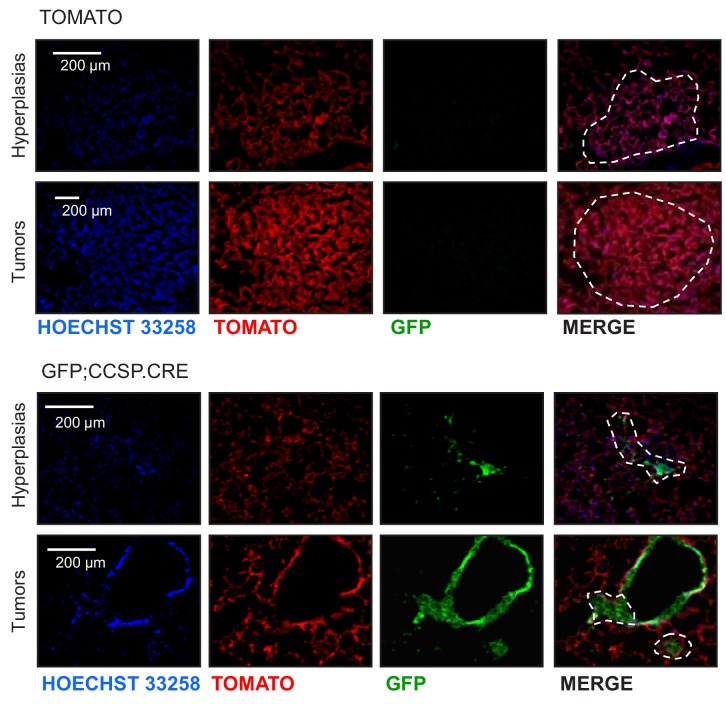
Genetic labeling of MCA/BHT-induced lung adenocarcinomas in two lineage reporter strains on the C57BL/6 background: representative images. Single-channel (endogenous TOMATO and GFP labeling and Hoechst 33258 nuclear stain) and merged images of lung hyperplasias and tumors (dashed outlines) of TOMATO and GFP;CCSP.CRE mice at six months after initiation of treatment with four weekly intraperitoneal injections of 15 mg/Kg 3-methyl-1,2-dyhydrobenzo[j]aceanthrylene (MCA) followed by eight weekly intraperitoneal injections of 200 mg/Kg butylated hydroxytoluene (BHT) (*n* = 8/group). Note the absence of GFP-labeling in lesions of TOMATO mice and the GFP-labeled lesions of GFP;CCSP.CRE mice.

**Figure 1—figure supplement 15.**
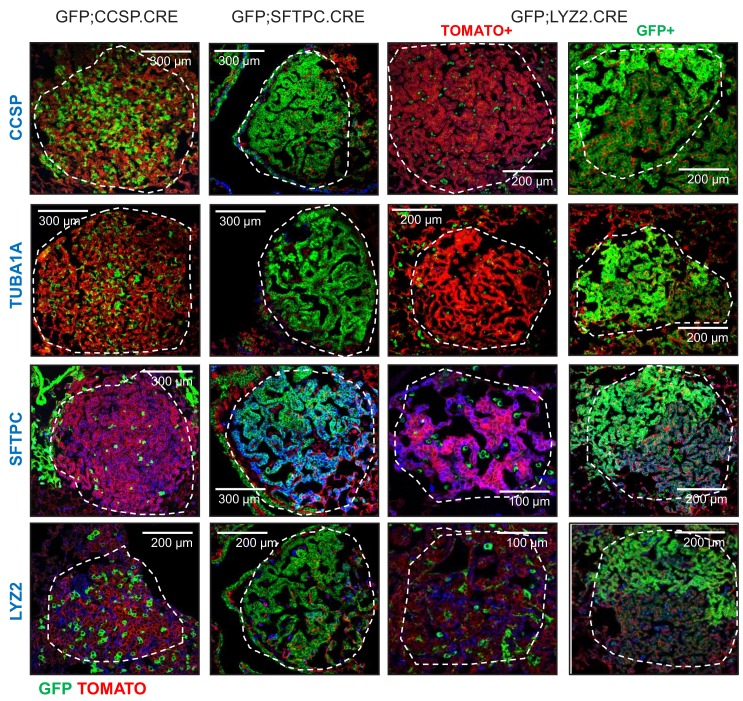
Protein marker expression of urethane-induced lung adenocarcinomas in three lineage-labeled mouse strains on the C57BL/6 background: representative images. Lineage marker protein-stained lung adenocarcinomas (dashed outlines) from genetically marked mice (*n* = 10/group). Note the genetic GFP-labeled tumor cells of GFP;CCSP.CRE mice that have lost CCSP and have acquired SFTPC with or without LYZ2 protein marker expression. CCSP, Clara cell secretory protein; TUBA1A, acetylated α-tubulin; SFTPC, surfactant protein C; LYZ2, lysozyme 2.

**Figure 1—figure supplement 16.**
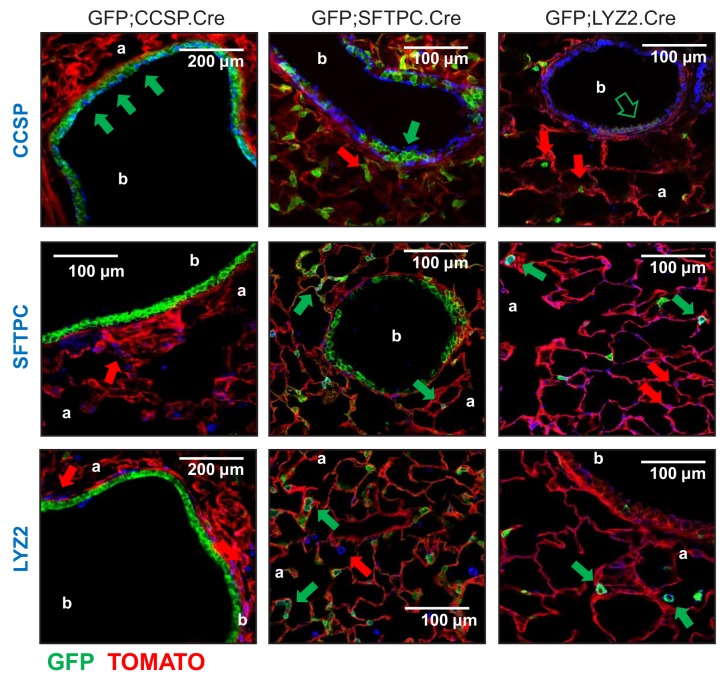
Genetic lineage labels of protein-marked cells in three lineage reporter strains on the FVB background: representative images. Representative merged fluorescent microscopic images from lineage marker-stained lung sections of 6-week-old lineage reporter mice (*n* = 5/group). Arrows indicate cells expressing the respective marker protein with (green) or without (red) genetic lineage-labeling. CCSP, Clara cell secretory protein; SFTPC, surfactant protein C; LYZ2, lysozyme 2; b, bronchi; a, alveoli.

**Figure 1—figure supplement 17.**
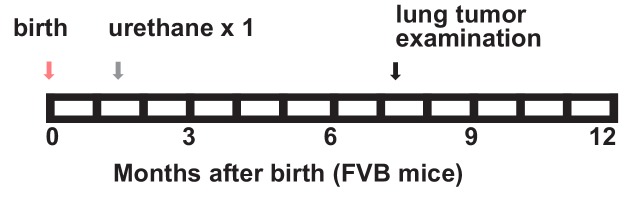
A single-hit mouse model for urethane-induced lung adenocarcinoma induction in naturally susceptible FVB mice. Schematic of single-hit urethane administration tailored to yield 100% tumor incidence in FVB mice: one intraperitoneal injection of 1 g/Kg urethane (ethyl carbamate, EC; gray arrow) is delivered at six weeks after birth (pink arrow) and lungs are examined six months later (black arrow).

**Figure 1—figure supplement 18.**
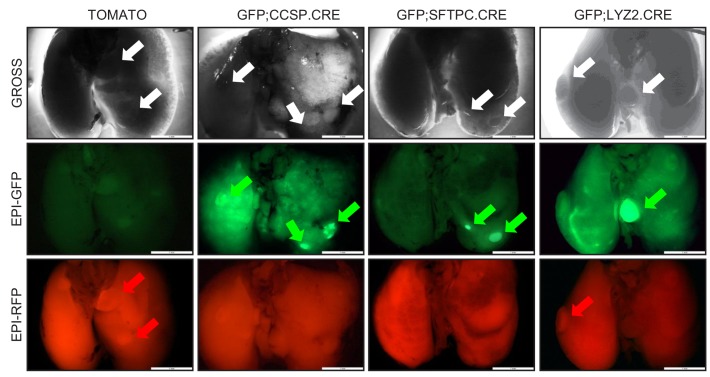
High-throughput epifluorescent detection of genetic labeling of urethane-induced lung adenocarcinomas in four lineage reporter strains on the FVB background: representative images. Representative photographs (top) and green (middle) and red (bottom) epifluorescence images of tumor-bearing lungs from genetically lineage-marked FVB mice at six months after a single intraperitoneal urethane injection (*n* ≥ 8/strain). Arrows indicate all (white), GFP-labeled (green), and TOMATO-labeled (red) lung tumors.

**Figure 1—figure supplement 19.**
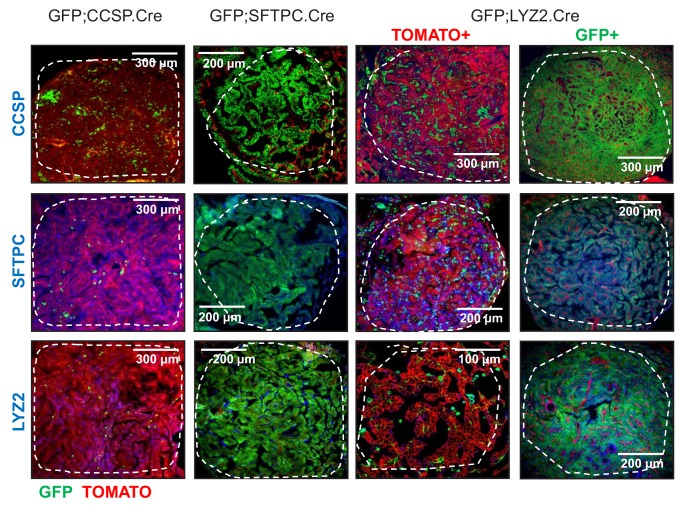
Genetic labeling of urethane-induced lung adenocarcinomas in three lineage reporter strains on the FVB background: representative images. Representative merged fluorescent microscopic images of lineage marker protein-stained lung tumors (dashed outlines) from genetically marked mice (FVB background) at six months after a single intraperitoneal urethane injection (*n* ≥ 10/strain). Note the genetic GFP-labeled tumor cells of GFP;CCSP.CRE mice that have lost CCSP and have acquired SFTPC with or without LYZ2 protein marker expression. CCSP, Clara cell secretory protein; SFTPC, surfactant protein C; LYZ2, lysozyme 2.

Here we aimed at identifying the cell lineage(s) that give rise to human-relevant tobacco carcinogen-triggered LUAD in mice and that regenerate adult murine alveoli after injury. For this, we combined mouse models of genetic labeling and ablation of airway and alveolar epithelial cells with noxious and tumorigenic insults to the adult lung. To achieve this, we adapted multi-hit chemical carcinogen exposure protocols to the murine C57BL/6 strain that is resistant to chemical tumor induction (Miller et al., 2003; Malkinson et al., 1997; Stathopoulos et al., 2007), and corroborated the findings with the FVB strain that is susceptible to single-hit carcinogenesis (Westcott et al., 2015; Stathopoulos et al., 2007; Vreka et al., 2018). We show that aging, toxic, and carcinogen insults to the adult mouse lung cause expansion of airway-marked cells to the alveolar parenchyma, where they express the alveolar marker SFTPC and facilitate alveolar repair and carcinogenesis. In addition, we report how airway cells preferentially sustain chemical-induced *KRAS* mutations leading to LUAD that are spatially linked with neighboring bronchi. Moreover, genetic ablation of airway cells is shown to hinder alveolar maintenance and carcinogenesis in mice, indicating a central role for these cells in alveolar regeneration and LUAD triggered in response to environmental challenges.

## Results

### Accurate genetic labeling of the airway lineage

To evaluate the contribution of different epithelial lung cell lineages to chemical-induced LUAD, we crossed a CRE-reporter strain that switches somatic cells from membranous tdTomato (mT; hereafter TOMATO) to membranous GFP (mG; hereafter GFP) fluorescence upon CRE-mediated recombination (mT/mG; hereafter TOMATO mice) (Muzumdar et al., 2007) to six different CRE-driver strains on the C57BL/6 background (Desai et al., 2014; Oikonomou et al., 2012; Okubo et al., 2005; Hayashi et al., 2002; Ogilvy et al., 1998; Tronche et al., 1999). This permitted the permanent genetic GFP*-*labeling of different lung cell lineages (mouse strains are listed in Figure 1A and Figure 1—figure supplement 2, and in Materials and methods and in Appendix 1). Double heterozygote offspring at six postnatal weeks (i.e., after mouse lung development is complete [Zuo et al., 2015; Desai et al., 2014]) were examined for GFP*-*labeling (results are shown in Figure 1A, Figure 1—figure supplements 3 and 4, and in Figure 1—figure supplement 4—source data 1). This approach labeled permanently all AEC of GFP;CCSP.CRE mice, some AEC and all ATII of GFP;SFTPC.CRE mice, some ATII and all AMΦ of GFP;LYZ2.CRE mice, and various other cells in the remaining intercrosses (Figure 1A, Figure 1—figure supplements 3–5, and Figure 1—figure supplement 5—source data 1). Co-localization of GFP*-*labeling with lineage protein markers (listed in Figure 1A and Figure 1—figure supplement 1) revealed that genetic GFP-labeling in GFP;CCSP.CRE mice marked all airway epithelial cells including club and ciliated cells, in GFP;SFTPC.CRE mice most airway and all alveolar epithelial type II cells, and in GFP;LYZ2.CRE mice some alveolar epithelial type II cells and all alveolar macrophages (Figure 1B, Figure 1—figure supplements 6–8, Figure 1—figure supplement 7—source data 1, Figure 1—figure supplement 8—source data 1).These findings show precise airway epithelial lineage labeling in GFP;CCSP.CRE mice and non-specific airway/alveolar/myeloid lineage labeling in GFP;SFTPC.CRE and GFP;LYZ2.CRE mice.

### Airway cells in chemical-induced lung adenocarcinoma

We next triggered LUAD in GFP;CCSP.CRE, GFP;SFTPC.CRE, and GFP;LYZ2.CRE mice on the C57BL/6 background using repetitive exposures to the tobacco carcinogens urethane (ethyl carbamate, EC; stand-alone mutagen and tumor promoter) (Westcott et al., 2015; Miller et al., 2003; Stathopoulos et al., 2007; Vreka et al., 2018) or 3-methylcholanthrene followed by butylated hydroxytoluene (MCA/BHT; a two-hit mutagen/tumor promoter regimen) (Malkinson et al., 1997) (Figure 1C, Figure 1—figure supplements 9 and 10, and Figure 1—figure supplement 10—source data 1). In both models, preneoplastic (airway epithelial hyperplasias and atypical alveolar hyperplasias) and neoplastic (adenoma and LUAD) lesions classified according to established guidelines (Nikitin et al., 2004) were located both in the airways and the alveolar regions. However, established lung tumors were most frequently located near or inside the airways (Figure 1C and Figure 1—figure supplement 11). All hyperplasias and tumors of GFP;SFTPC.CRE and some of GFP;LYZ2.CRE mice were GFP-labeled, but this was not informative, since baseline marking of GFP;SFTPC.CRE and GFP;LYZ2.CRE mice were non-specific. Interestingly, all hyperplasias and tumors of GFP;CCSP.CRE mice contained GFP-labeled airway cells that did not express the club cell marker CCSP anymore, but had acquired expression of the alveolar epithelial markers SFTPC with or without LYZ2 (Figure 1D, Figure 1—figure supplements 12–15, and Figure 1—figure supplement 13—source data 1). Identical results were recapitulated using single urethane hits to GFP;CCSP.CRE, GFP;SFTPC.CRE, and GFP;LYZ2.CRE mice backcrossed >F12 to the susceptible FVB strain, which result in human LUAD-like mutations including *Kras*^Q61R^ (Westcott et al., 2015; Vreka et al., 2018; Kanellakis et al., 2019) (Figure 1D and Figure 1—figure supplements 16–19). Collectively, these data support that airway cells contribute to chemical-induced LUAD, shifting from airway to alveolar marker expression during carcinogenesis.

### Airway cells sustain *Kras*^Q61R^ mutations and give rise to juxtabronchial tumors

**Figure 2.**
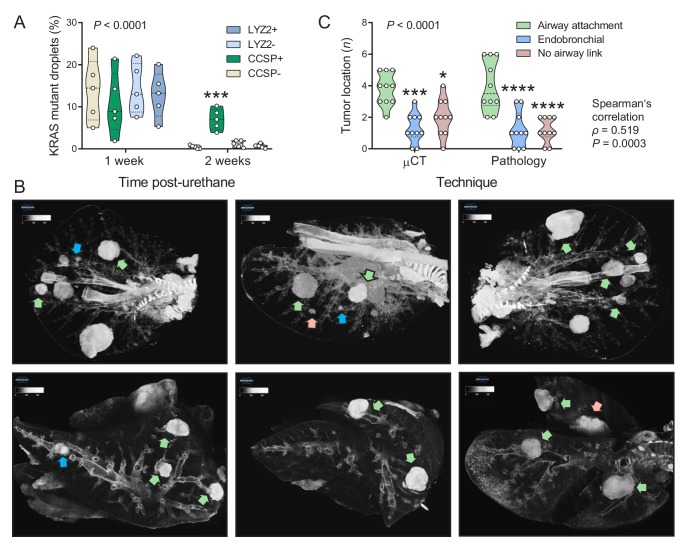
Airway cells sustain *Kras*^Q61R^ mutations inflicted by urethane and give rise to juxtabronchial lung adenocarcinomas. (**A**) DNA was extracted from the lungs of GFP;CCSP.CRE and GFP;LYZ2.CRE mice (FVB strain) one and two weeks post-urethane treatment (*n* = 5/group). Summary of duplexed digital droplet PCR (ddPCR) results using primers and probes specific for the *Rosa^mT^* and the *Kras*^WT^ sequences. Note that all cell types equally suffer initial *Kras*^Q61R^ mutations, but only GFP-labeled cells of GFP;CCSP.CRE mice (i.e. airway cells) maintain the *Kras*^Q61R^ mutation after two weeks. See also Figure 2—figure supplement 1. Data are shown as violin plot. *P*, overall probability, two-way ANOVA. ***: p<0.001 compared with all other groups, Bonferroni post-tests. (**B**) Representative high-resolution micro-computed tomography (μCT) lung sections (top) and three-dimensional reconstructions (bottom) from urethane-treated FVB mice six months after treatment (*n* = 10). Note lung tumors attached to (green arrows) or contained within (blue arrows) the airways, as well as lung tumors with no obvious link to a bronchus (red arrows). (**C**) Summary of results from μCT (data from Figure 2B) and pathology (data from Figure 1C) shown as violin plot. *P*, probability, two-way ANOVA.*, ***, and ****: p<0.05, p<0.001, and p<0.0001, respectively, compared with airway-attached tumors, Bonferroni post-tests. Shown are also Spearman’s correlation coefficient (*ρ*) and probability (**P**) for correlation of μCT and pathology results. 10.7554/eLife.45571.031Figure 2—source data 1.Quantification of*Kras*mutant droplets in duplexed digital droplet PCR (ddPCR). 10.7554/eLife.45571.032Figure 2—source data 2.Quantification of tumor airway link.

We next used digital droplet PCR (ddPCR) to determine the lung lineages that suffer *Kras*^Q61R^ driver mutations at early time-points after single urethane hits (Westcott et al., 2015; Vreka et al., 2018; Kanellakis et al., 2019). For this, GFP;CCSP.CRE and GFP;LYZ2.CRE mice backcrossed >F12 to the susceptible FVB strain received urethane and duplexed ddPCR designed to single-copy-co-amplify *Kras* and *Rosa^mT^* was performed one and two weeks later. Interestingly, GFP-labeled cells of both mouse strains had *Kras*^Q61R^ mutations at one week post-urethane, but *Kras*^Q61R^ mutations selectively persisted in GFP-labeled airway cells in the lungs of GFP;CCSP.CRE mice at two weeks (Figure 2A, Figure 2—figure supplement 1, and Figure 2—source data 1). In addition, three-dimensional reconstruction of tumor-bearing lungs of FVB mice at 6 months post-urethane using high-resolution micro-computed tomography (μCT) revealed that most lung tumors were spatially linked with the airways, in accord with pathology results (Figure 2B and C, and Figure 2—source data 2). These results support the involvement of airway cells in chemical-induced lung adenocarcinoma formation in mice.

**Figure 2—figure supplement 1.**
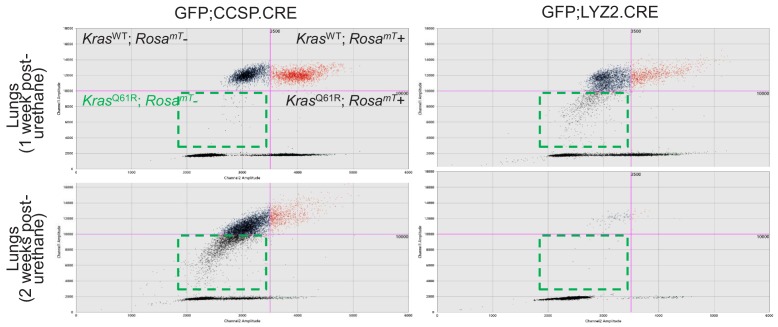
Airway cells sustain *Kras*^Q61R^ mutations inflicted by urethane. DNA was extracted from the lungs of GFP;CCSP.CRE and GFP;LYZ2.CRE mice (FVB strain) one and two weeks post-urethane treatment (*n* = 5/group). Representative gating strategy of digital droplet PCR (ddPCR) using primers and probes specific for the *Rosa^mT^* and the *Kras*^WT^ sequences. Dashed outlines indicated GFP+*Kras*^Q61R^+ droplet gates.

### Alveolar dissemination of airway-labeled cells during carcinogenesis

**Figure 3.**
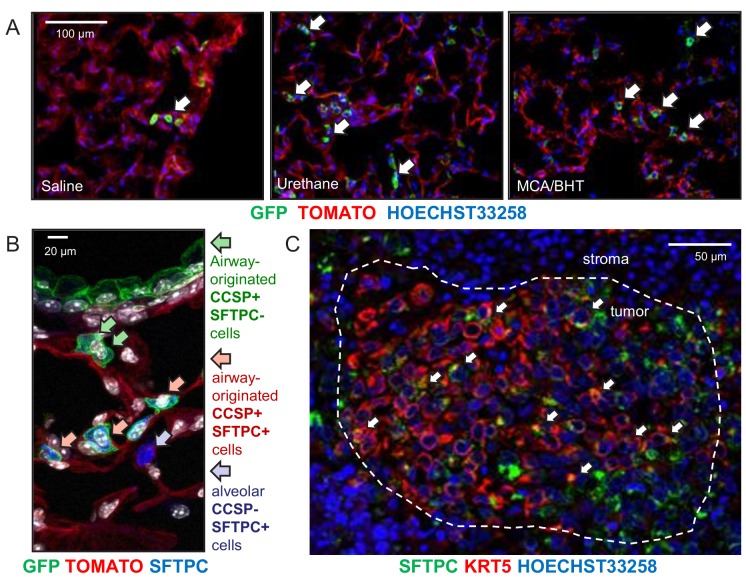
Expansion of airway cells in the tumor-initiated lung. (**A**) Non-neoplastic alveolar regions from lung sections of saline-, urethane (ethyl carbamate, EC)-, and 3-methyl-1,2-dyhydrobenzo[j]aceanthrylene/butylated hydroxytoluene (MCA/BHT)-treated GFP;CCSP.CRE mice at six months into treatment (*n* = 8 mice/group). Note the few GFP-labeled cells of saline-treated mice and their increased numbers in carcinogen-treated mice (arrows). See also Figure 3—figure supplements 1 and 2. (**B**) Juxtabronchial region from lung section of urethane-treated GFP;CCSP.CRE mouse at six months into treatment (*n* = 22) stained for the alveolar type II cell marker SFTPC. Arrows and legend indicate different phenotypes of extrabronchial GFP-labeled cells. See also Figure 3—figure supplements 3–5. (**C**) Merged high-power image of SFTPC and KRT5 co-staining of human lung adenocarcinoma (*n* = 10) shows significant co-localization of the two markers in a subset of tumor cells (arrows). See also Figure 3—figure supplement 6. CCSP, Clara cell secretory protein; SFTPC, surfactant protein C; KRT5, keratin 5.

Since airborne carcinogens act globally on the respiratory field (Franklin et al., 1997), we examined non-neoplastic alveolar areas of carcinogen-treated GFP;CCSP.CRE mice, to discover markedly increased numbers of GFP-labeled cells in the alveoli of carcinogen-treated mice compared with saline-treated or naïve controls (Figure 3A, Figure 3—figure supplements 1 and 2, and Figure 3—figure supplement 2—source data 1). Immunostaining revealed that juxtabronchial GFP-labeled cells still expressed CCSP, but lost CCSP and acquired SFTPC expression when located in alveoli and tumors (Figure 3B and Figure 3—figure supplements 3 and 4). The expansion of airway cells after urethane exposure was also documented using bioluminescent imaging of double heterozygote offspring of CCSP.CRE intercrosses with Luciferase-expressing (LUC) mice (Safran et al., 2003), a strain emitting light specifically from airway epithelia (Figure 3—figure supplement 5, and Figure 3—figure supplement 5—source data 2). In addition, co-staining of human LUAD (Giopanou et al., 2015) for the alveolar marker SFTPC and the airway markers CCSP and KRT5 showed co-localization of SFTPC with KRT5 but not with CCSP (Figure 3C and Figure 3—figure supplement 6). These results suggest that airway epithelial cells expand to alveolar regions during field cancerization by tobacco carcinogens, a process involving either direct alveolar cell recycling by airway epithelial cells or transient CCSP expression by alveolar cells during carcinogenesis. Moreover, that human and murine LUAD carry airway imprints although their location and protein expression suggests an alveolar origin (Desai et al., 2014; Aberle et al., 2011; Mason et al., 2000; Lindskog et al., 2014; Sutherland and Berns, 2010).

**Figure 3—figure supplement 1.**
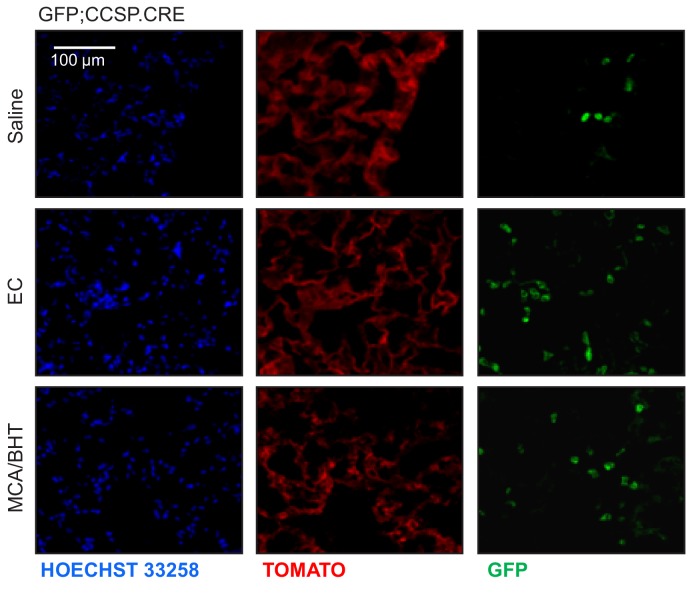
Airway-labeled cells in the alveoli of carcinogen-exposed C57BL/6 mice: representative images. Single-channel microscopy images (endogenous TOMATO and GFP labeling with Hoechst 33258 nuclear stain) of non-neoplastic alveolar regions of GFP;CCSP.CRE mice treated as in Figure 3A.

**Figure 3—figure supplement 2.**
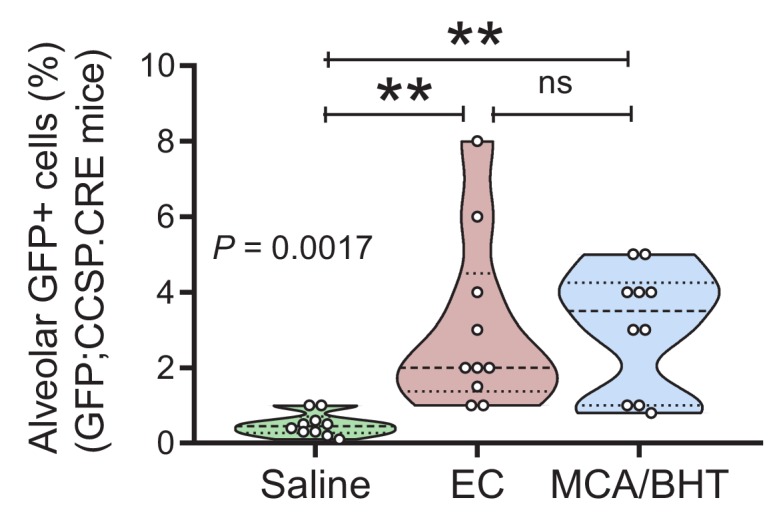
Airway-labeled cells in the alveoli of carcinogen-exposed C57BL/6 mice: data summary. Data summary (shown as violin plot) from GFP;CCSP.CRE mice treated as in Figure 3A (*n* = 10/group). *P*, overall probability, one-way ANOVA. ns and **: p>0.05 and p<0.01 for the indicated comparisons, Bonferroni post-tests. 10.7554/eLife.45571.040Figure 3—figure supplement 2—source data 1.Quantification of alveolar GFP+ cells in GFP;CCSP.CRE mice after carcinogen hit.

**Figure 3—figure supplement 3.**
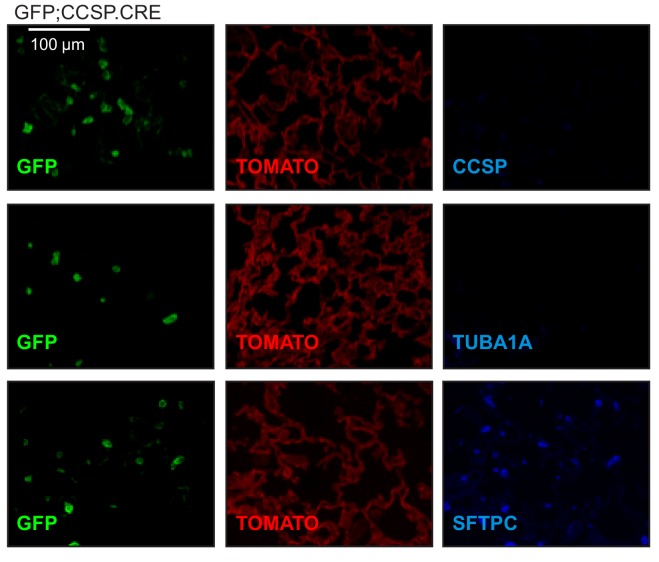
Airway-labeled cells in the alveoli of carcinogen-exposed mice express SFTPC. Single-channel images of non-neoplastic distal lung regions of urethane-treated GFP;CCSP.CRE mice at six months into treatment (*n* = 22), stained for the lung cell markers Clara cell secretory protein (CCSP), acetylated α-tubulin (TUBA1A), and surfactant protein C (SFTPC). Note the genetic GFP-labeled tumor cells that have lost CCSP and have acquired SFTPC protein marker expression.

**Figure 3—figure supplement 4.**
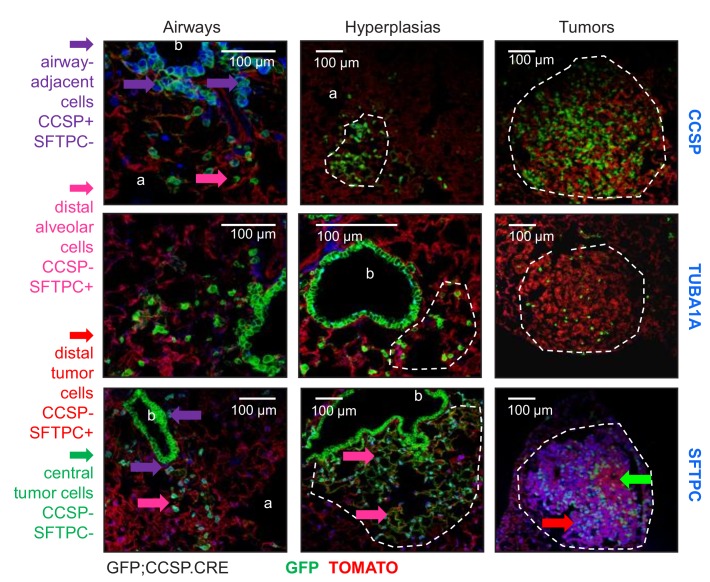
Airway-labeled cells in environmental-induced lung tumors express SFTPC. Juxtabronchial regions, alveolar hyperplasias, and tumors (dashed lines) of lungs from urethane-treated GFP;CCSP.CRE mice at six months into treatment (*n* = 22) stained for lineage marker proteins Clara cell secretory protein (CCSP), acetylated α-tubulin (TUBA1A), and surfactant protein C (SFTPC). Arrows and legend indicate different phenotypes of extrabronchial GFP-labeled cells. a, alveoli; b, bronchi.

**Figure 3—figure supplement 5.**
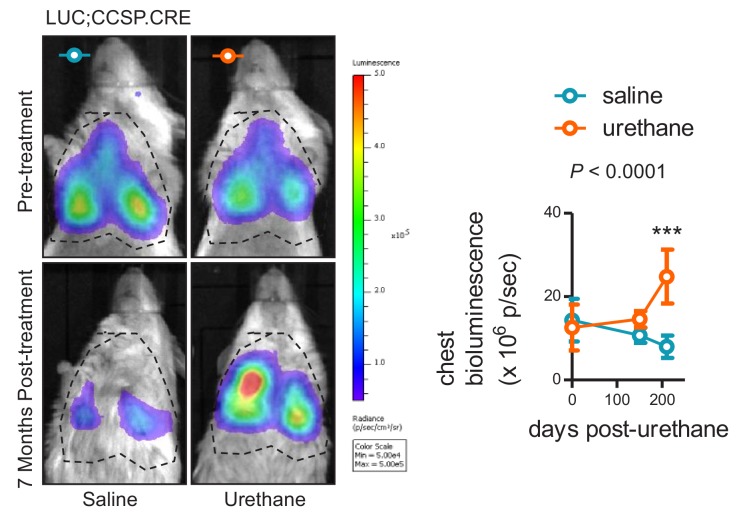
In vivo bioluminescent detection of the airway lineage in the lungs of saline- and carcinogen-treated mice. Representative merged bioluminescence/photographic images (left) and data summary (right) of LUC;CCSP.CRE mice (FVB background) before and seven months after saline (one intraperitoneal injection of 100 µL; *n* = 6) or urethane (one intraperitoneal injection of 1 g/Kg in 100 µL saline; *n* = 5) treatment. Note that in this model light is emitted exclusively by genetically CCSP-labeled cells over the lungs. Note also the signal decrease in saline- and increase in urethane-treated mice. Data are given as mean ± SD. *P*, overall probability, two-way ANOVA. ***: p<0.001 for comparison with saline, Bonferroni post-test. 10.7554/eLife.45571.041Figure 3—figure supplement 5—source data 2.Quantification of chest bioluminescence signal in LUC;CCSP.CRE mice after urethane exposure.

**Figure 3—figure supplement 6.**
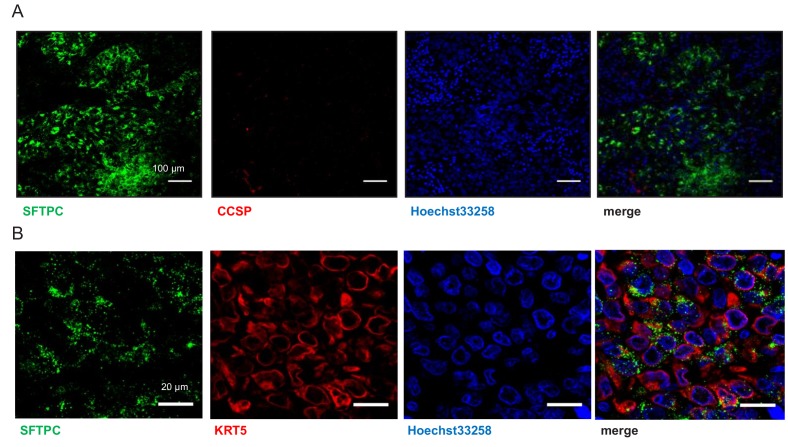
Human lung adenocarcinomas co-express airway and alveolar markers. Co-staining of human lung adenocarcinomas for SFTPC and either CCSP (A; *n* = 10) or KRT5 (B; *n* = 10) shows absence of CCSP expression and significant co-localization of SFTPC and KRT5 in a subset of tumor cells. CCSP, Clara cell secretory protein; KRT5, keratin 5; SFTPC, surfactant protein C.

### Airway cells in the aging and injured adult alveolus

**Figure 4.**
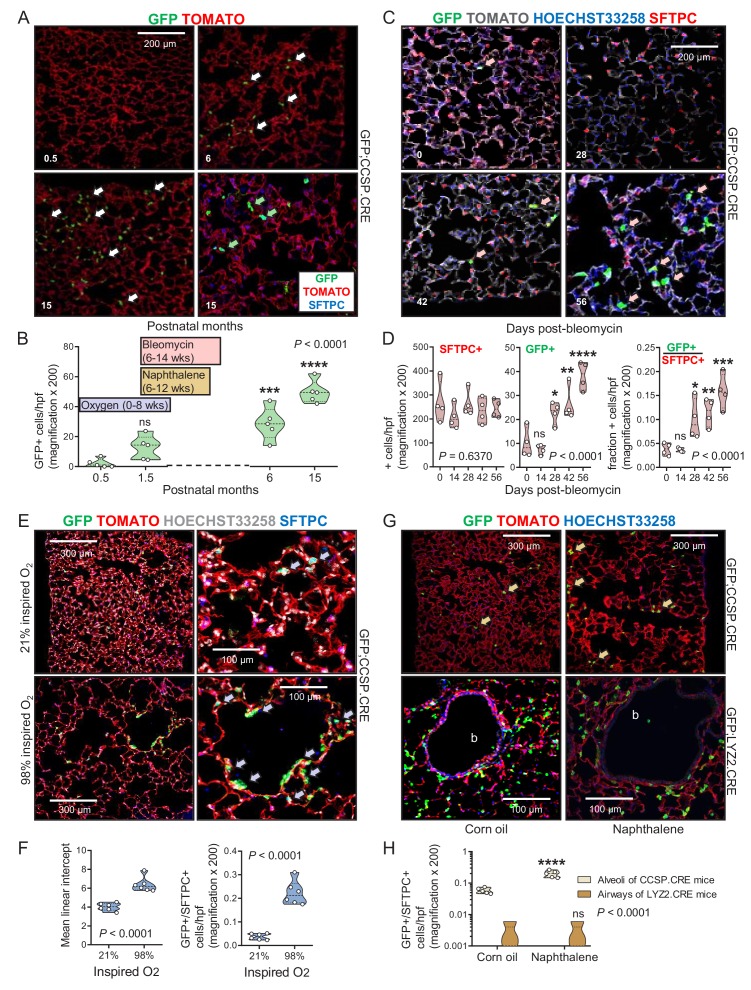
Airway cells in alveolar repair. (**A**) Non-neoplastic alveolar regions from lung sections of aging GFP;CCSP.CRE mice (bottom right section is also SFTPC-immunostained) show increasing numbers of alveolar GFP-labeled cells with age (arrows). Green arrows: genetically GFP-labeled, SFTPC-immunoreactive airway cells in alveolus of 15-month-old GFP;CCSP.CRE mouse. (**B**) Data summary (*n* = 5 mice/time-point) from (**A**) shown as violin plot. Color-coded boxes indicate time windows of experiments in (**C-H**). *P*, probability, one-way ANOVA. ns, ***, and ****: p>0.05, p<0.001, and p<0.0001, respectively, for comparison with time-point zero by Bonferroni post-tests. (**C**) SFTPC-immunostained lung sections of GFP;CCSP.CRE mice show accelerated increase of alveolar GFP-labeled SFTPC-immunoreactive airway cells after bleomycin treatment (arrows). See also Figure 4—figure supplement 1 and Figure 4—figure supplement 2. (**D**) Data summary from (**C**) shown as violin plots (*n* = 4 mice/time-point). *P*, probabilities, one-way ANOVA. ns, *, **, ***, and ****: p>0.05, p<0.05, p<0.01, p<0.001, and p<0.0001, respectively, for comparison with day zero by Bonferroni post-tests. (**E**) SFTPC-stained lung sections of GFP;CCSP.CRE mice at two months after perinatal exposure to 98% O_2_ show enlarged alveoli (evident by increased mean linear intercept) enriched in GFP-labeled SFTPC-immunoreactive airway cells (arrows) compared with 21% O_2_. (**F**) Data summary from (**E**) shown as violin plots (*n* = 6 mice/group). *P*, probabilities, t-test. (**G**) Lung sections (top) of GFP;CCSP.CRE mice (*n* = 5 mice/group) show enrichment of alveoli in GFP-labeled cells post-naphthalene treatment (arrows). Lung sections (bottom) of GFP;LYZ2.CRE mice (*n* = 5 mice/group) at six weeks post-naphthalene show no bronchial (**b**) GFP-labeled cells. See also Figure 4—figure supplements 3 and 4. (**H**) Data summary from (**G**) shown as violin plot (*n* = 5 mice/time-point). *P*, probability, two-way ANOVA. ns and ****: p>0.05 and p<0.0001, respectively, for comparison with corn oil by Bonferroni post-tests. CCSP, Clara cell secretory protein; SFTPC, surfactant protein C; LYZ2, lysozyme 2. 10.7554/eLife.45571.047Figure 4—source data 1.Quantification of alveolar GFP+ cells in GFP;CCSP.CRE mice during aging. 10.7554/eLife.45571.048Figure 4—source data 2.Quantification of SFTPC+ and GFP+ cells in GFP;CCSP.CRE mice after bleomycin treatment. 10.7554/eLife.45571.049Figure 4—source data 3.Data of mean linear intercept and GFP+/SFTPC+cells in GFP;CCSP.CRE mice after hyperoxia treatment. 10.7554/eLife.45571.050Figure 4—source data 4.Data of GFP+/SFTPC+ cells in GFP;CCSP.CRE and GFP;LYZ2.CRE mice after naphthalene treatment.

We next examined the kinetics of lineage-labeled cells during aging, injury, and repair. While the number of GFP-labeled cells in the alveoli of aging GFP;SFTPC.CRE and GFP;LYZ2.CRE mice was stable, GFP-labeled airway cells in the alveoli of aging GFP;CCSP.CRE mice progressively increased and expressed SFTPC protein (Figure 4A and B and Figure 4—source data 1). Bleomycin treatment, which depletes alveolar type II cells (Lawson et al., 2005), accelerated the accumulation of GFP-labeled airway cells in the alveoli and in urethane-triggered LUAD (Figure 4C and D, Figure 4—figure supplements 1 and 2, Figure 4—source data 2, and Figure 4—figure supplement 2—source data 1). GFP-labeled airway cells expressing the alveolar marker SFTPC also increased in the alveoli of GFP;CCSP.CRE mice exposed to perinatal hyperoxia that damages forming alveoli (Rawlins et al., 2009), and in the alveoli of GFP;CCSP.CRE mice treated with naphthalene that kills airway epithelial cells (Sutherland and Berns, 2010; Rawlins et al., 2009), but were not identified within the airways of naphthalene-treated GFP;CCSP.CRE mice; these appeared to be repopulated by GFP-labeled airway cells that express the club cell marker CCSP (Figure 4E–4H, Figure 4—figure supplements 3 and 4, Figure 4—source datas 3 and 4, and Figure 4—figure supplement 4—source data 2). In line with the latter finding, no GFP-labeled alveolar cells were identified in the airways of GFP;LYZ2.CRE mice recovering from naphthalene-induced injury (Figure 4G and H). Taken together, the data indicate that airway-originated cells repopulate both the airways and the alveoli during aging and recovery from injury, while alveolar cells do not reconstitute the airways, in line with previous findings (Desai et al., 2014; Rawlins et al., 2009). The observed alveolar spread of airway-labeled cells was explained by either peripheral migration of airway cells or transient CCSP expression by regenerating alveolar cells.

**Figure 4—figure supplement 1.**
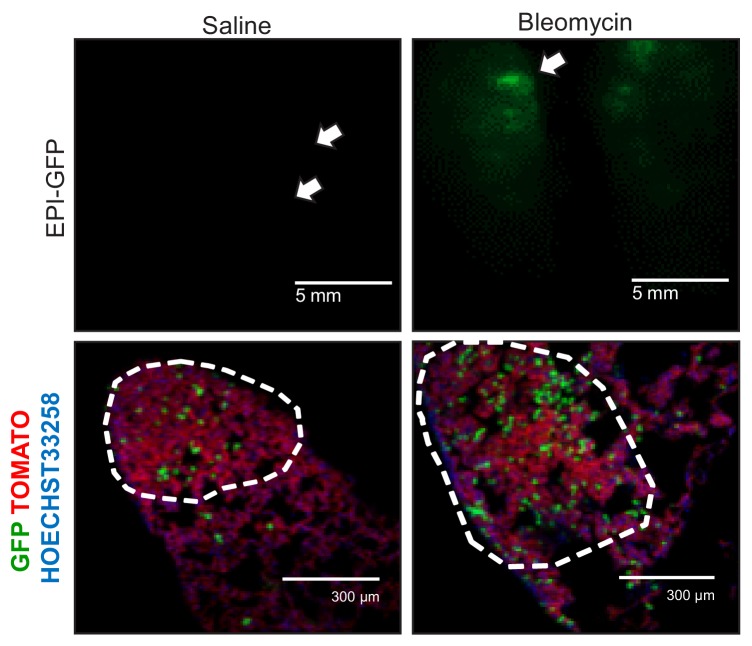
Alveolar type II cell ablation using bleomycin pre-treatment increases airway-labeled cells in urethane-induced lung tumors: representative images. Representative epifluorescence (top) and merged fluorescent microscopy (bottom) images of tumor-bearing lungs and lung tumors of six-week-old GFP;CCSP.CRE mice that received intratracheal saline or 0.08 units bleomycin (*n* = 6/group), were allowed to recover for one month, and subsequently received ten weekly intraperitoneal injections of 1 g/Kg urethane to be sacrificed six months after the first urethane injection. Arrows and dashed outlines indicate lung tumors.

**Figure 4—figure supplement 2.**
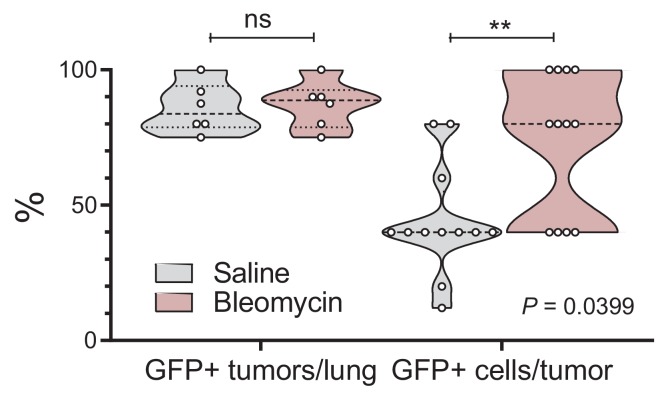
Alveolar type II cell ablation using bleomycin pre-treatment increases airway-labeled cells in urethane-induced lung tumors: data summary. Violin plot of GFP-labeled tumors/mouse (*n* = 6 mice/group) and GFP-labeled cells/tumor (*n* = 12 tumors/group; *n* = 2 tumors/mouse were examined) from experiment described in Figure 4—figure supplement 1. Note the enrichment of lung adenocarcinomas in GFP-labeled cells in response to bleomycin, which depletes resident alveolar type II cells. *P*, overall probability, two-way ANOVA. **: p<0.01 for comparison with saline, Bonferroni post-test. 10.7554/eLife.45571.051Figure 4—figure supplement 2—source data 1.Quantification of GFP+ tumors/lung and GFP+ cells/tumor in GFP;CCSP.CRE mice after bleomycin and urethane treatment.

**Figure 4—figure supplement 3.**
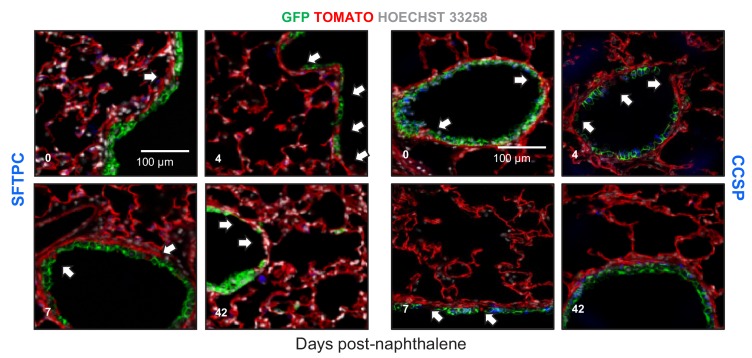
Airway epithelial cell ablation using naphthalene is restored by airway-labeled cells: representative images. Representative fluorescent microscopic images of lungs of GFP;CCSP.CRE mice at different time-points after intraperitoneal injection of 250 mg/Kg naphthalene given at six weeks of age. Shown are merges of Hoechst 33258-stain, endogenous TOMATO- and GFP-labeling, and immunostains for surfactant protein C (SFTPC, left) or Clara cell secretory protein (CCSP, right). Arrows denote naphthalene-induced airway epithelial gaps that are restored by GFP-labeled airway cells that express CCSP, but not SFTPC protein.

**Figure 4—figure supplement 4.**
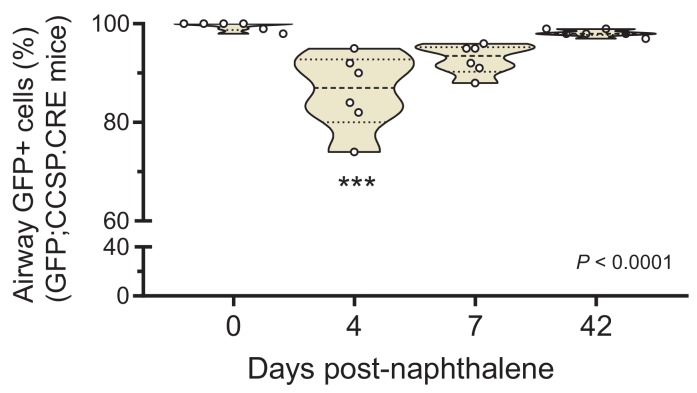
Airway epithelial cell ablation by naphthalene: data summary. Violin plot of percentage of GFP-labeled airway cells from experiment described in Figure 4—figure supplement 3 (*n* = 6 mice/time-point). *P*, overall probability, one-way ANOVA. ***: p<0.001 for the comparison with day zero, Bonferroni post-test. 10.7554/eLife.45571.052Figure 4—figure supplement 4—source data 2.Quantification of GFP+ airway cells in GFP;CCSP.CRE mice after naphthalene treatment.

### Airway cells maintain alveoli and foster tumors

**Figure 5.**
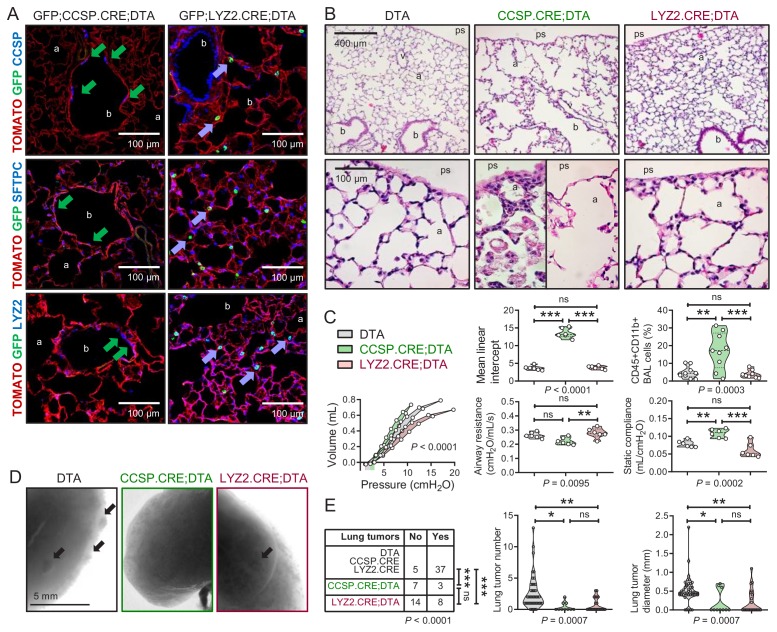
Airway cell-ablated mice display alveolar destruction and are protected from carcinogenesis. (**A**) Lineage marker-immunostained lung sections of 12-week-old GFP;CCSP.CRE;DTA and GFP;LYZ2.CRE;DTA mice (*n* = 6/group) show increased bronchial and alveolar size and flat CCSP+ SFTPC+ LYZ2+ cells in the airways of GFP;CCSP.CRE;DTA mice (green arrows), and CCSP-SFTPC-LYZ2+ alveolar macrophages in the airspaces of GFP;LYZ2.CRE;DTA mice (blue arrows). See also Figure 5—figure supplements 1 and 2. (**B**) Hematoxylin and eosin-stained lung sections (*n* = 6/group) from 12-week-old DTA (controls), CCSP.CRE;DTA (airway epithelial suicide model), and LYZ2.CRE;DTA (alveolar epithelial suicide model) mice. (**C**) Data summaries of mean linear intercept, bronchoalveolar lavage (BAL) myeloid cells, pressure-volume curves, airway resistance, and static compliance (*n* = 6–10/group) from 12-week-old DTA, CCSP.CRE;DTA, and LYZ2.CRE;DTA mice shown as violin plots. *P*, probabilities, one-way ANOVA. ns, **, and ***: p>0.05, p<0.01, and p<0.001, respectively, for the indicated comparisons, Bonferroni post-tests. (**D**) Lung photographs of control, CCSP.CRE;DTA, and LYZ2.CRE;DTA mice at six months into treatment with urethane started at six weeks of age. (**E**) Incidence table and data summaries of lung tumors from (**D**) (violin plots; *n* is given in table). *P*, probabilities, χ^2^-test (table) and one-way ANOVA (graphs). ns, *, **, and ***: p>0.05, p<0.05, p<0.01, and p<0.001, respectively, for the indicated comparisons, Fischer’s exact tests (table) or Bonferroni post-tests (graphs). a, alveoli; b, bronchi; ps, pleural space; v, vessel. CCSP, Clara cell secretory protein; SFTPC, surfactant protein C; LYZ2, lysozyme 2. 10.7554/eLife.45571.056Figure 5—source data 1.Quantifications of data shown in Figure 5C. 10.7554/eLife.45571.057Figure 5—source data 2.Quantifications of data shown in Figure 5D and E.

To further examine the role of airway and alveolar cells in alveolar homeostasis and lung carcinogenesis, we ablated them by crossing CCSP.CRE, SFTPC.CRE, and LYZ2.CRE mice to mice expressing Diphtheria toxin in somatic cells upon CRE-mediated recombination (DTA mice) (Voehringer et al., 2008). Triple transgenic GFP;DRIVER.CRE;DTA intercrosses were also generated to evaluate ablation efficiency. As expected, SFTPC.CRE;DTA and GFP;SFTPC.CRE;DTA mice were fetal lethal (no double or triple heterozygote offspring was obtained by *n* > 3 intercrosses,>10 litters, and >60 off-springs for each genotype; p<0.0001, Fischer’s exact test). However, all other ablated mice survived till adulthood. Airway epithelial ablation was complete in GFP;CCSP.CRE;DTA mice, while some GFP-labeled alveolar macrophages persisted in GFP;LYZ2.CRE;DTA mice, presumably freshly recruited monocytes initiating LYZ2 expression. Immunostaining revealed that the denuded airway epithelium of 12-week-old GFP;CCSP.CRE;DTA mice contained few flat CCSP+SFTPC+LYZ2+ immunoreactive cells, while the apparently intact alveolar spaces of GFP;LYZ2.CRE;DTA mice harbored only some CCSP-SFTPC-LYZ2+immunoreactive alveolar macrophages (Figure 5A, Figure 5—figure supplements 1 and 2, and Figure 5—figure supplement 2—source data 1). Remarkably, morphometric and functional analyses of 12-week-old DTA control, CCSP.CRE;DTA, and LYZ2.CRE;DTA mice showed that LYZ2.CRE;DTA mice displayed normal airway caliper and mean linear intercept (measures of airway and alveolar structure), normal number of CD45+ CD11b+ myeloid cells in bronchoalveolar lavage (BAL; measure of airspace inflammation), and normal airways resistance and static compliance (measures of airway and alveolar function) compared with DTA controls. However, CCSP.CRE;DTA mice displayed widened airway and alveolar dimensions with inflammatory interalveolar septal destruction evident by increased mean linear intercept, CD45+ CD11b+ cells in BAL, and static compliance (Figure 5B and C and Figure 5—source data 1), mimicking human chronic obstructive pulmonary disease (Barnes et al., 2015). Finally, we exposed control and ablated mice to ten consecutive weekly urethane exposures. All mice survived six months into carcinogen treatment, and CCSP.CRE;DTA and LYZ2.CRE;DTA mice were equally protected from LUAD development compared with controls (Figure 5D and E, and Figure 5—source data 2). Taken together, these results show that the CCSP+ airway lineage maintains postnatal alveolar structure and function, and, together with the LYZ2+ alveolar lineage, are required for lung adenocarcinoma development.

**Figure 5—figure supplement 1.**
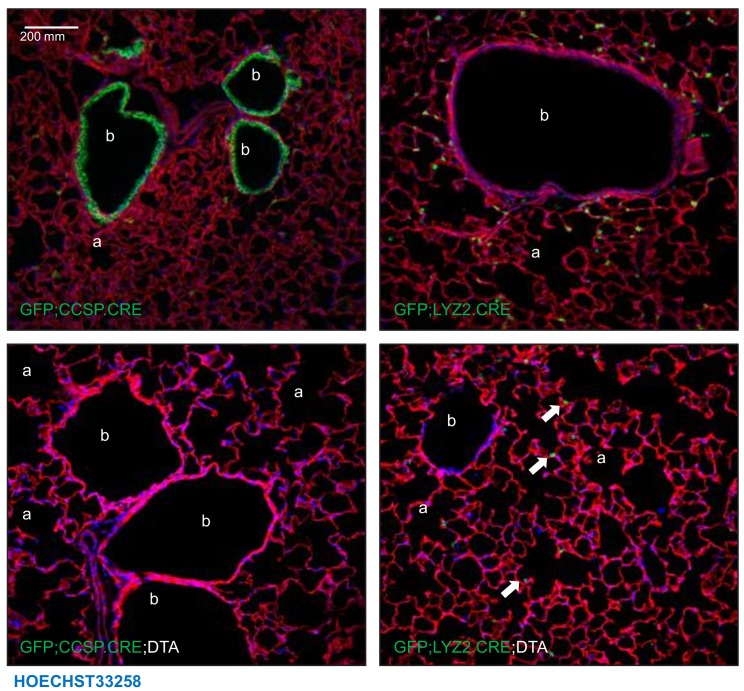
Triple transgenic mouse models for validation of genetic pulmonary lineage ablation: representative images. Representative lung sections of 12-week-old GFP;CCSP.CRE, GFP;LYZ2.CRE, GFP;CCSP.CRE;DTA, and GFP;LYZ2.CRE;DTA mice (*n* = 6/group). Shown are merges of Hoechst 33258-stained endogenous TOMATO- and GFP-labeling. Note increased bronchial (**b**) and alveolar (**a**) size, complete airway epithelial denudement, and prominent distortion of bronchial and alveolar structure of GFP;CCSP.CRE;DTA mice compared with other strains, mimicking chronic obstructive pulmonary disease. Note also the presence of some GFP-labeled alveolar macrophages in GFP;LYZ2.CRE;DTA mice (arrows). a, alveoli; b, bronchi.

**Figure 5—figure supplement 2.**
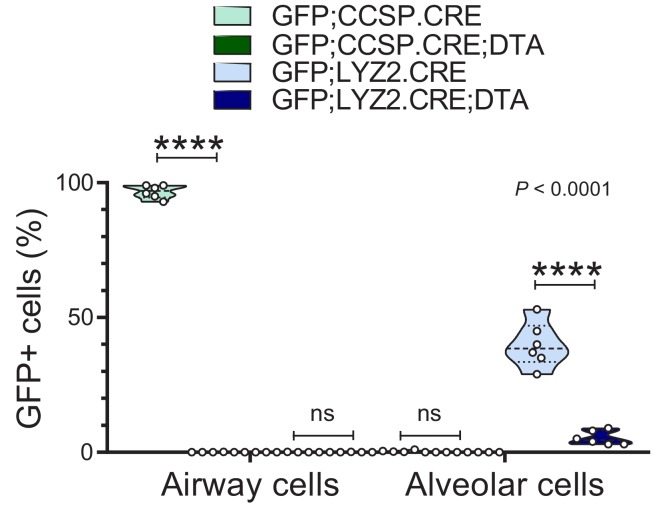
Triple transgenic mouse models for validation of genetic pulmonary lineage ablation: data summary. Violin plot of GFP-labeling of lung sections of 12-week-old mice from Figure 5—figure supplement 1 (*n* = 6/group). Note the complete ablation of airway cells in GFP;CCSP.CRE mice and the persistence of some GFP-labeled alveolar macrophages in GFP;LYZ2.CRE;DTA mice. Measurements were from at least five non-overlapping tumor, airway, or alveolar fields/lung. *P*, overall probability, two-way ANOVA. ns and ****: p>0.05 and p<0.0001, respectively, for the indicated comparisons by Bonferroni post-tests. 10.7554/eLife.45571.058Figure 5—figure supplement 2—source data 1.Data of GFP+ cells in airways and alveoli of GFP;CCSP.CRE, GFP;CCSP.CRE;DTA, GFP;LYZ2.CRE and GFP;LYZ2.CRE;DTA mice.

### Airway epithelial signatures in experimental and human lung adenocarcinoma

**Figure 6.**
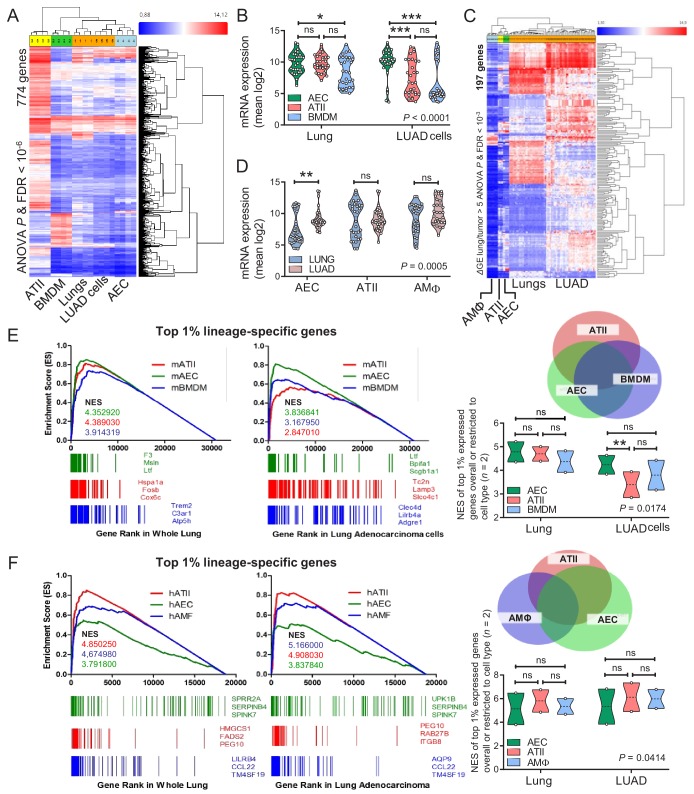
Airway and alveolar signatures in murine and human lung adenocarcinoma (LUAD). (**A, B**) RNA of mouse urethane-induced LUAD cell lines, lungs obtained pre- and one week post-urethane treatment, airway epithelial cells (AEC), alveolar type II cells (ATII), and bone marrow-derived macrophages (BMDM) was examined by Affymetrix Mouse Gene ST2.0 microarrays (*n* = 4/group). (**A**) Heat map of genes significantly differentially expressed (overall ANOVA and FDR p<10^−6^) shows accurate hierarchical clustering. (**B**) Expression of the 30 top-represented transcripts of AEC, ATII, and BMDM in lungs and LUAD cells. See also Figure 6—figure supplements 1–4. (**C, D**) RNA of human LUAD (*n* = 40), never-smoker lung tissue (*n* = 30), primary AEC (*n* = 5), primary ATII (*n* = 4), and alveolar macrophages (AMΦ; *n* = 9) was analyzed by Affymetrix Human Gene ST1.0 microarrays. (**C**) Heat map of genes significantly differentially expressed (*Δ*GE > 5 fold) between LUAD and lung (ANOVA and FDR p<10^−3^) shows accurate hierarchical clustering. (**D**) Mean expression levels of the 30 top-represented transcripts of human AEC, ATII, and AMΦ in lungs and LUAD. (**E, F**) Gene set enrichment analyses, including normalized enrichment scores (NES), of mouse (**E**) and human (**F**) AEC, ATII, and BMDM/AMΦ signatures (defined as the top 1% expressed genes overall or exclusive to the cell type; *n* = 2) in mouse and human LUAD transcriptomes shows significant enrichment of the AEC (but not the ATII and BMDM/AMΦ) signature compared with lung (nominal p<0.0001 for all, family-wise error rates FWER <0.01). Gene symbols indicate the top three lagging genes from each signature and shows loss of *Scgb1a1* (encoding CCSP) by LUAD. See also Figure 6—figure supplements 5 and 6. Data are given as violin plots. *P*, two-way ANOVA probabilities. ns, *, **, and ***: p>0.05, p<0.05, p<0.01, and p<0.001 for the indicated comparisons by Bonferroni post-tests. ANOVA, analysis of variance; FDR, false discovery rate. 10.7554/eLife.45571.066Figure 6—source data 1.Cross-examination of signature genes of murine AEC, ATII cells, BMDM, LUAD cells and lungs. 10.7554/eLife.45571.067Figure 6—source data 2.Cross-examination of signature genes of human AEC, ATII cells, BMDM, LUAD cells and lungs. 10.7554/eLife.45571.068Figure 6—source data 3.Quantification of gene set enrichment analyses data shown in Figure 6E. 10.7554/eLife.45571.069Figure 6—source data 4.Quantification of gene set enrichment analyses data shown in Figure 6F.

We subsequently examined the transcriptomes of cell lines isolated from urethane-induced LUAD (Kanellakis et al., 2019) and of murine lungs with those of murine AEC isolated from tracheal explants, of murine ATII cells (Frank et al., 2016), and of murine bone-marrow-derived macrophages (BMDM). The AEC transcriptome was specifically enriched in LUAD cells compared with whole lungs (Figure 6A and B, Figure 6—figure supplement 1, and Figure 6—source data 1). LUAD cell lines lost expression of epithelial markers compared with their native lungs, but displayed up-regulated expression of LUAD markers (i.e., *Krt18* and *Krt20*), of epidermal growth factor receptor ligands (*Areg* and *Ereg*), and of the *Myc* oncogene (Figure 6—figure supplements 2–4, and Figure 6—figure supplement 2—source data 1). Similar analyses of the transcriptomes of human LUAD and corresponding healthy lungs (Kabbout et al., 2013), and of primary human AEC, ATII, and AMΦ (Clark et al., 2015; Dancer et al., 2015; Lee et al., 2009) also disclosed that the AEC transcriptome was significantly enriched in LUAD compared with healthy lungs (Figure 6C and D and Figure 6—source data 2). Gene set enrichment analyses (GSEA) showed that the mouse AEC transcriptome predominated over ATII/BMDM transcriptomes in LUAD cells (Figure 6E, Figure 6—figure supplement 5, and Figure 6—source data 3). In addition, the human AEC transcriptome was enriched equally with ATII/AMΦ transcriptomes in human LUAD compared with healthy lungs (Figure 6F, Figure 6—figure supplement 6, and Figure 6—source data 4). These results showed the presence of an anticipated alveolar and an unexpected airway epithelial transcriptomic signature in tobacco carcinogen-induced LUAD of mice and men. The more pronounced results in mice were plausible by the early nature of the human surgical specimens examined compared with our murine cell lines that present advanced metastatic tumor cells.

**Figure 6—figure supplement 1.**
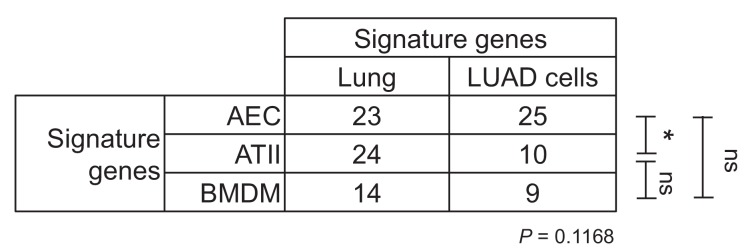
Lineage-specific gene expression in mouse lung adenocarcinoma cell lines induced by urethane compared with mouse lungs. RNA of mouse urethane-induced lung adenocarcinoma (LUAD) cell lines, lungs obtained pre- and one week post-urethane treatment, and airway epithelial cells (AEC), alveolar type II cells (ATII), and bone marrow-derived macrophages (BMDM) was examined by Affymetrix Mouse Gene ST2.0 microarrays (*n* = 4/group). Shown is the number of genes out of the 30 top-represented transcripts of AEC, ATII, and BMDM within the top-2000-expressed genes of lungs and LUAD cells.

**Figure 6—figure supplement 2.**
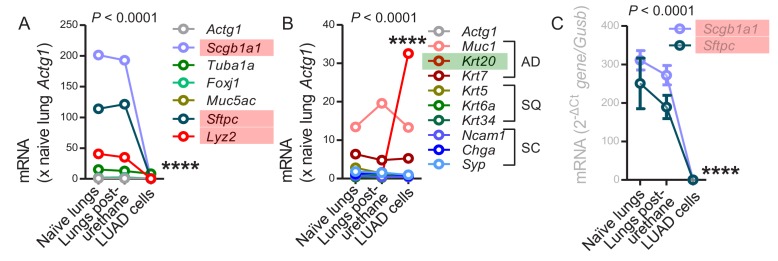
Loss of lineage marker expression in mouse lung adenocarcinoma cell lines induced by urethane. Mean expression levels of selected transcripts, including lineage markers and markers of histologic subtype in lung adenocarcinoma (LUAD) cell lines compared with lungs pre- and one week post-urethane treatment (A and B, microarrays from Figure 6—figure supplement 1, *n* = 2/group; C, qPCR, *n* = 3/group). AD, adenocarcinoma; SQ, squamous cell carcinoma; SC, small cell carcinoma. *P*, overall probability, two-way ANOVA. ****: p<0.0001 for the highlighted genes compared with lungs (red, significantly down-regulated; green, significantly up-regulated). 10.7554/eLife.45571.070Figure 6—figure supplement 2—source data 1.Quantification of gene expression levels of data shown in Figure 6—figure supplement 2.

**Figure 6—figure supplement 3.**
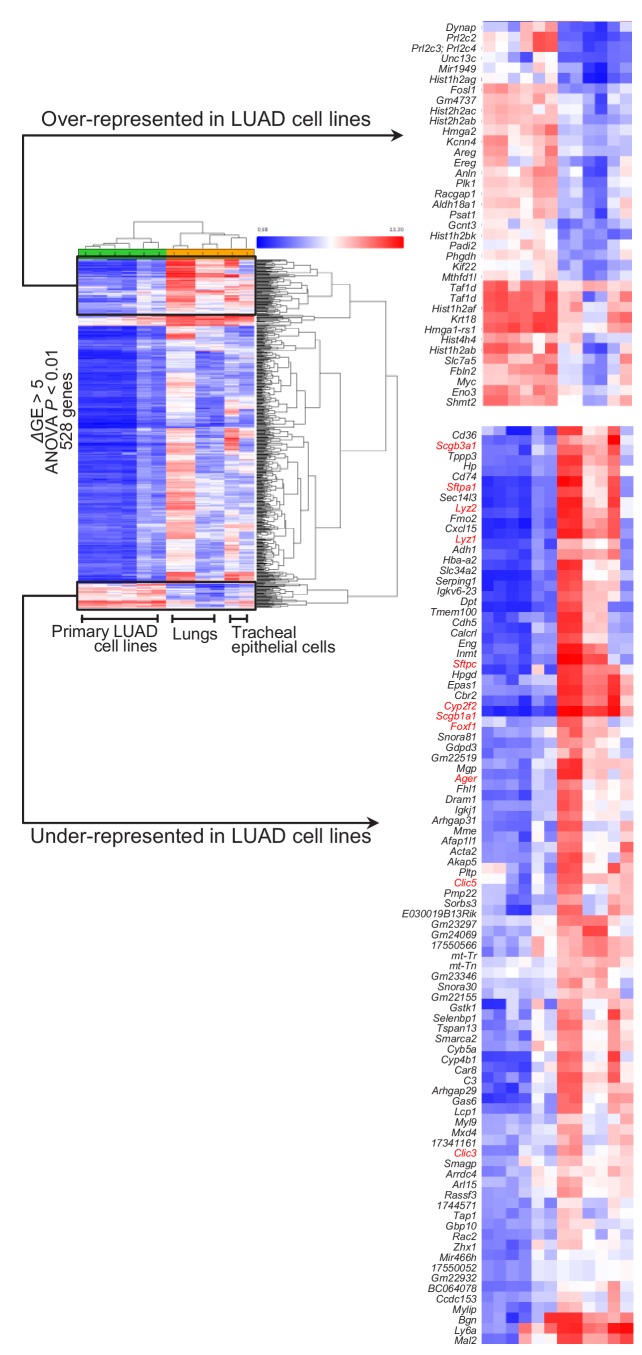
Loss of lineage marker expression in mouse lung adenocarcinoma cell lines induced by urethane compared with mouse lungs: heat maps. 528 genes differentially expressed between six different lung adenocarcinoma cell lines cultured from urethane-induced lung tumors and six benign respiratory mouse samples, including lungs of saline- and urethane-treated mice obtained at one week post-treatment, as well as primary mouse tracheal epithelial cells using the cut-offs indicated. Whole heat map (left) showing the accurate hierarchical clustering of the samples according to differentially expressed genes, as well as the top over- and under-represented genes (right). Note the universal loss of expression of lineage markers by lung adenocarcinoma cells (genes in red font). ANOVA, analysis of variance; FDR, false discovery rate.

**Figure 6—figure supplement 4.**
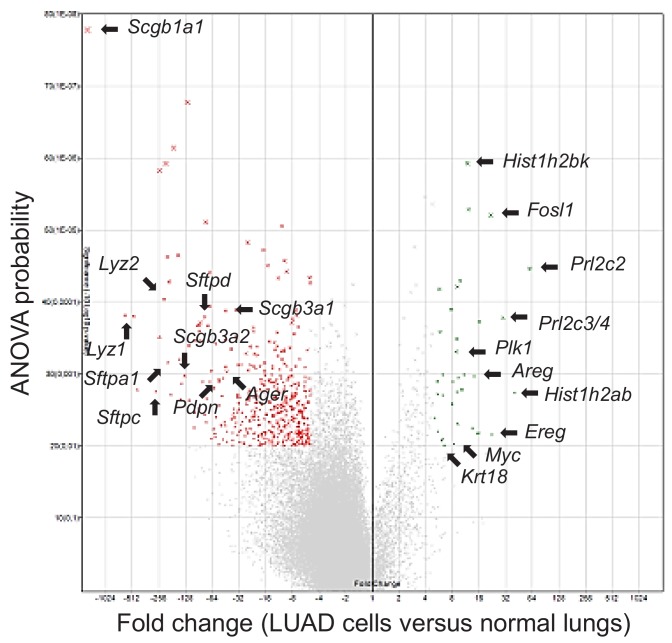
Loss of lineage marker expression in mouse lung adenocarcinoma cell lines induced by urethane compared with mouse lungs: volcano plot. Shown are selected top over- and under-represented genes (arrows) from microarrays from Figure 6—figure supplement 2.

**Figure 6—figure supplement 5.**
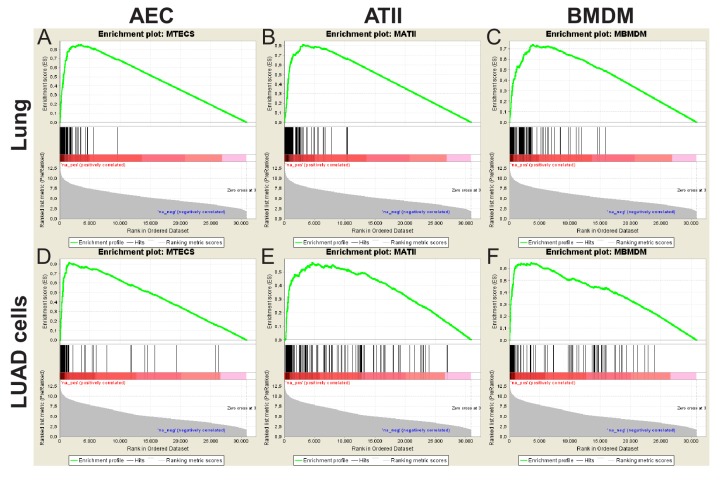
Mouse gene set enrichment analyses. Shown are gene set enrichment analyses of airway epithelial cell (AEC), alveolar type II cell (ATII), and bone marrow-derived macrophage (BMDM) transcriptome signatures in mouse lungs (top) and urethane-induced lung adenocarcinoma (LUAD) cell lines (bottom) transcriptomes. The data were used to design Figure 6E.

**Figure 6—figure supplement 6.**
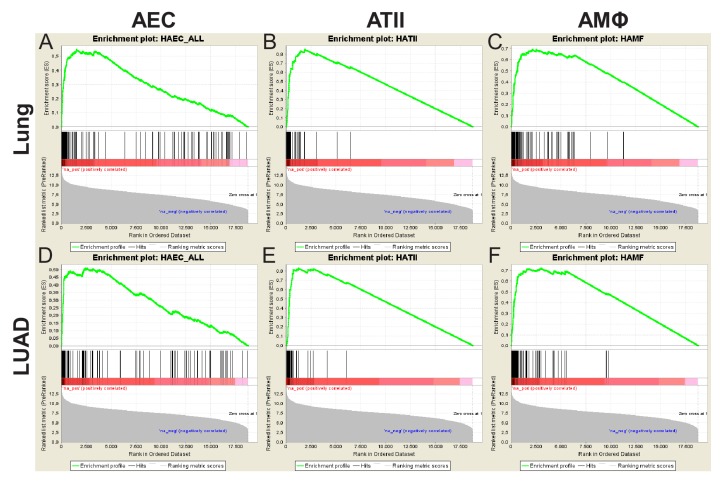
Human gene set enrichment analyses. Affymetrix Human Gene ST1.0 microarrays hybridized with RNA of human lung adenocarcinomas (LUAD; *n* = 40), never-smoker lung tissues (*n* = 30), primary airway epithelial cells (AEC; *n* = 5), primary alveolar type II cells (ATII; *n* = 4), and alveolar macrophages (AMΦ; *n* = 9) were cross-examined. Shown are gene set enrichment analyses of AEC, ATII, and AMΦ signatures in lung (top) and LUAD (bottom) transcriptomes. The data were used to design Figure 6F.

## Discussion

**Figure 7.**
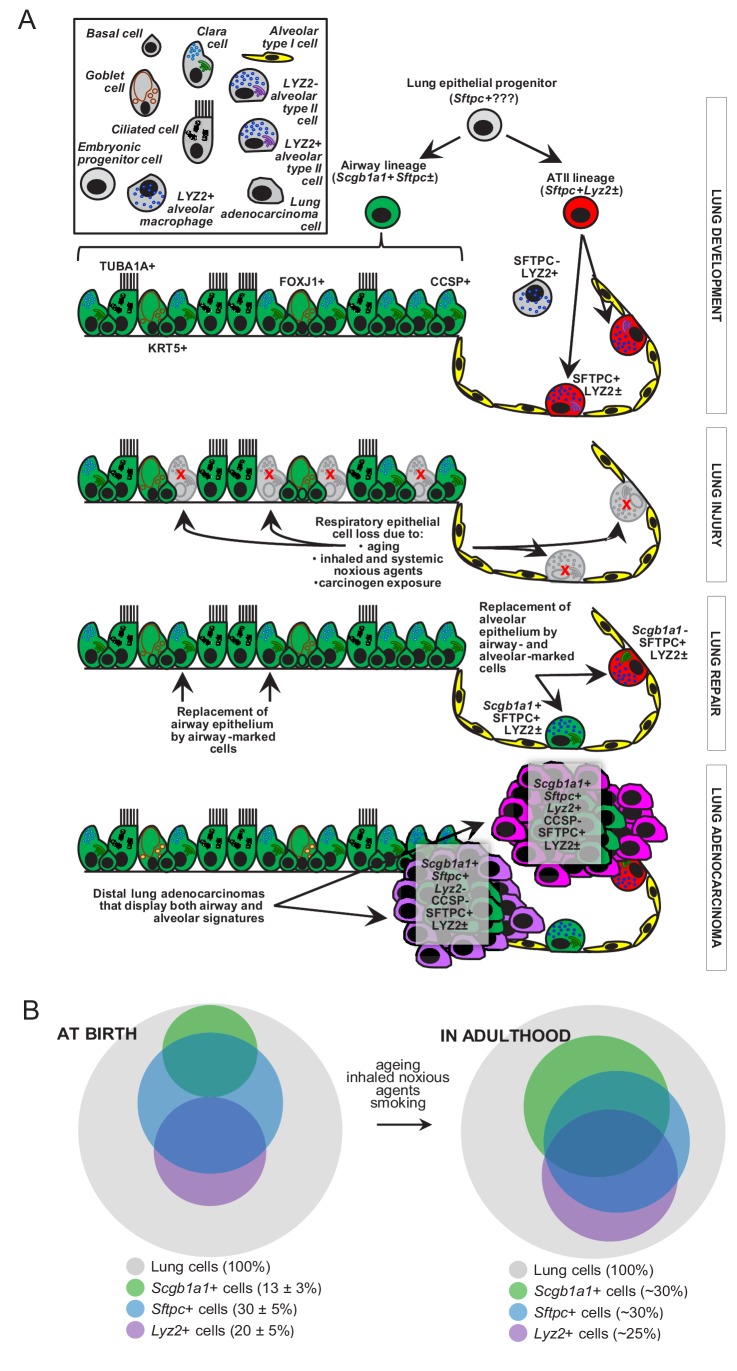
Proposed role of airway-marked cells in murine lung maintenance and adenocarcinoma. (**A**) Our evidence supports the existence of distinct developmental ancestries for airway epithelial (AEC) and alveolar type II (ATII) cells, notwithstanding their common descent from an early (possibly *Sftpc*+) lung epithelial progenitor. The developmental airway lineage (*Scgb1a1+ Sftpc*±; green) gives rise to all types of airway cells, including club, ciliated, goblet, basal, and other cells, while the developmental ATII lineage (*Sftpc+ Lyz2*±; red) gives rise to ATII cells before birth. These lineages appear to be segregated in the growing unaffected lung of the mouse till the age of six weeks, which roughly corresponds to a human age of six years, where cellular proliferation in the human lungs ceases. Thereafter, and likely due to the continuous exposure of the lungs to inhaled noxious agents, gradual expansion of *Scgb1a1+ Sftpc*± marked cells ensues. Upon lung injury, this process is accelerated. Similarly, during carcinogenesis caused by chemical tobacco smoke carcinogens, *Scgb1a1+ Sftpc*± marked cells expand and are ubiquitously present in peripheral lung adenocarcinomas. (**B**) Proposed neonatal proportions and postnatal dynamics of pulmonary epithelial cells during adulthood. Estimated proportions of lineage-marked cells at birth, based on flow cytometry and co-localization of proteinaceous and genetic cell marking. Lung lineages appear to be segregated in the growing lung till the age of full lung development (six weeks in mice and 6–8 years in humans) or till lung injury ensues. Schematic of proposed postnatal redistribution of marked cells in the adult lung. Upon injury, during multi-stage field carcinogenesis, or even during unchallenged aging, *Scgb1a1*+ marked cells appear in the distal alveolar regions, thereby maintaining lung structure and function. Bubble size indicates relative marked cell abundance. CCSP, Clara cell secretory protein; FOXJ1, forkhead box J1; KRT5, keratin 5; LYZ2, lysozyme 2; SFTPC, surfactant protein C; TUB1A1, acetylated α-tubulin.

We characterized the dynamics of respiratory epithelial cells in the postnatal mouse lung during aging and after challenge with noxious and carcinogenic insults. The contributions of airway cells to chemical-induced lung adenocarcinoma are described for the first time (Figure 7A). Although the peripheral location and molecular phenotype of murine and human lung adenocarcinoma (i.e., the expression of the alveolar epithelial marker SFTPC) suggest an alveolar origin, we show here that both airway and alveolar cells are found in environmental-induced lung adenocarcinoma and that, in fact, airway cells may play a more prominent role during the initial steps of carcinogenesis. Furthermore, airway cells are implicated in postnatal alveolar maintenance during aging and recovery from injury. Our analyses facilitate insights into the dynamics of epithelial lineages in the postnatal lung (Figure 7B) and indicate that airway cells are essential for the sustained structural and functional integrity of adult alveoli. Finally, mouse and human lung adenocarcinomas are shown to bare transcriptome markings of highly enriched airway signatures, rendering our findings plausible in both experimental and human lung adenocarcinoma.

This study addresses the cellular and molecular signatures of chemical-induced lung adenocarcinoma. Lung tumors induced in two different mouse strains by two different chemical regimens contained in tobacco smoke are shown to contain airway epithelial markings. This is important because human lung adenocarcinoma is inflicted by chronic exposure to tobacco smoke and other environmental exposures (Hecht, 1999; Campbell et al., 2016; Cancer Genome Atlas Research Network, 2014; Westcott et al., 2015; Miller et al., 2003; Malkinson et al., 1997; Alexandrov et al., 2016; Castelletti et al., 2019). As such, the mutation profile of the human disease is more closely paralleled by chemical-induced murine lung tumors compared with lung cancers triggered by transgenic expression of *Kras*^G12C^ or *Kras*^G12D^ in the respiratory epithelium (Westcott et al., 2015). Although the latter transgenic tumors have been extensively studied (Kim et al., 2005; Cho et al., 2011; Xu et al., 2012; Sutherland et al., 2014; Mainardi et al., 2014; Desai et al., 2014), chemical-induced lung adenocarcinomas have not been investigated. In all mouse models we studied, all tumors contained the airway genetic marking, in contrast with the LYZ2 alveolar genetic marking which was dispensable for lung adenocarcinoma development. Our observations support the multi-stage field concept of chemical carcinogenesis (Franklin et al., 1997), according to which tumor-initiated cells undergo multiple steps of genomic evolution and phenotypic appearance that include an obligatory airway-like stage. In fact, the prevalence of a different *Kras* mutation in urethane-induced tumors (*Kras*^Q61R^) compared to *KRAS*^G12C/D^ mutations in the transgenic mouse models has led to the suggestion that chemical carcinogens introduce *KRAS* mutations in a different population of tumor-initiating cells than mouse models of genetic *KRAS* activation (Westcott et al., 2015). Our findings of airway epithelial cells being more sensitive than alveolar type II cells to *Kras*^Q61R^ mutations during the initial steps of urethane-induced carcinogenesis further supports this notion and render airway cells an attractive novel target for premalignancy.

The consistent finding of CCSP genetic markings (indicative of airway epithelial origin) together with SFTPC and LYZ2 protein expression (indicative of alveolar epithelial phenotype) in chemical-triggered lung adenocarcinomas and their precursor lesions implies three different scenarios for lung adenocarcinoma formation: i) airway epithelial cells colonize the distal lung during carcinogenesis thereby activating obligate (SFTPC+) and dispensable (LYZ2+) alveolar transcriptomes; ii) alveolar cells transit through an obligate CCSP+ with or without a dispensable LYZ2+ stage during the process; or iii) lung adenocarcinoma arises from multipotent progenitors that express multiple epithelial markers, such as those found during pulmonary embryogenesis, in human lung adenocarcinoma, and in other chronic lung diseases (Desai et al., 2014; Frank et al., 2016; Xu et al., 2016). However, in our view, the propensity of airway cells to survive *KRAS* mutations during early carcinogenesis, the close airway-proximity of lung tumors revealed by μCT and histology, as well as the fact that CCSP-labeled cells did not express the CCSP marker anymore, support a bronchial origin of these tumors. This view is in line with recent evidence for tobacco smoke-induced epigenetic changes that sensitize human airway epithelial cells to a single *KRAS* mutation (Vaz et al., 2017). Along these lines, the split genetic markings of chemical-induced lung adenocarcinomas of GFP;LYZ2.CRE mice indicates that LYZ2-labeled alveolar cells are dispensable for environmental lung adenocarcinoma, as opposed to what was previously shown for genetically-triggered lung adenocarcinoma (Desai et al., 2014).

Our approach focused on the integral assessment of changes in lung epithelial kinetics and transcriptome signatures during aging, injury, and carcinogenesis. The perpetual cell labeling approach we adopted was preferred over pulsed lineage tracing models because of the unprecedented accuracy of our CCSP.CRE strain in exclusively and completely labeling airway epithelial cells at the conclusion of development, allowing tracking of subsequent changes in adulthood. The identification of transcriptional programs that are activated during lung repair and carcinogenesis are of great importance for lung biology and are likely to lead to therapeutic innovations (Nagel et al., 2016). To this end, insertions and deletions in lineage-restricted genes were recently shown to occur in human lung adenocarcinoma (Imielinski et al., 2017). Moreover, integrin β_3_ and TANK-binding kinase one partner with oncogenic *KRAS* signaling to mediate cancer stemness and drug resistance (Barbie et al., 2009; Seguin et al., 2014). Along these lines, our findings of the involvement of airway epithelial cells in lung maintenance, repair, and carcinogenesis imply that at least some of these cells present lung stem cells with regenerative and malignant potential and thus marked therapeutic targets. This was evident in our hands by the facts that airway epithelial cells could maintain adult injured alveoli and sustain *KRAS* mutations induced by urethane.

In conclusion, airway cells contribute to alveolar maintenance and lung carcinogenesis in response to environmental challenges. Since defective epithelial repair underlies the pathogenesis of chronic lung diseases and since abundantly transcribed genes are central to the mutational processes that cause cancer, this finding is of potential therapeutic importance for chronic pulmonary diseases and lung cancer.

## Materials and methods

**Key resources table:** 

Reagent type (species) or resource	Designation	Source or reference	Identifiers	Additional information
Strain, strain background (*Mus musculus*)	C57BL/6	Jackson Laboratory	Stock #: 000664; RRID:IMSR_JAX:000664	
Strain, strain background (*M. musculus*)	FVB	Jackson Laboratory	Stock #: 001800; RRID:IMSR_JAX:001800	
Genetic reagent (*M. musculus*)	TOMATO	Jackson Laboratory	Stock #: 007676; RRID:IMSR_JAX:007676	Muzumdar et al., 2007
Genetic reagent (*M. musculus*)	LUC	Jackson Laboratory	Stock #: 005125; RRID:IMSR_JAX:005125	Safran et al., 2003
Genetic reagent (*M. musculus*)	DTA	Jackson Laboratory	Stock #: 009669; RRID:IMSR_JAX:009669	Voehringer et al., 2008
Genetic reagent (*M. musculus*)	LYZ2.Cre	Jackson Laboratory	Stock #: 004781; RRID:IMSR_JAX:004781	PMID: 10621974
Genetic reagent (*M. musculus*)	SOX2.Cre	Jackson Laboratory	Stock #: 008454; RRID:IMSR_JAX:008454	Hayashi et al., 2002
Genetic reagent (*M. musculus*)	VAV.Cre	Jackson Laboratory	Stock #: 008610; RRID:IMSR_JAX:008610	Ogilvy et al., 1998
Genetic reagent (*M. musculus*)	NES.Cre	Jackson Laboratory	Stock #: 003771; RRID:IMSR_JAX:003771	Tronche et al., 1999
Genetic reagent (*M. musculus*)	CCSP.Cre	European Mouse Mutant Archive	Stock #: EM:04965; RRID:IMSR_M231009	Oikonomou et al., 2012
Genetic reagent (*M. musculus*)	SFTPC.Cre	Mouse Genome Informatics	RRID:MGI:3574949	Okubo et al., 2005
Cell line (*M. musculus*)	LUAD cells	PMID: 30828726		Derived from urethane models
Biological sample (*Homo sapiens*)	Lung adenocarcinomas	Giopanou et al., 2015		Archival samples of patients with LUAD
Antibody	rabbit poyclonal anti-PCNA	Abcam	Cat. #: ab2426; RRID:AB_303062	IHC (1:3000)
Antibody	rabbit monoclonal anti-LYZ2	Abcam	Cat. #: ab108508; RRID:AB_10861277	IF (1:50)
Antibody	rabbit polyclonal anti-KRT5	Abcam	Cat. #: ab53121; RRID:AB_869889	IF (1:200)
Antibody	rabbit polyclonal anti-SFTPC	Santa Cruz Biotechnology	Cat. #: sc-13979; RRID:AB_2185502	IF (1:200)
Antibody	rabbit polyclonal anti-CCSP	Santa Cruz Biotechnology	Cat. #: sc-25555; RRID:AB_2269914	IF (1:200)
Antibody	goat polyclonal anti-CCSP	Santa Cruz Biotechnology	Cat. #: sc-9772; RRID:AB_2238819	IF (1:1000)
Antibody	mouse monoclonal anti-acetylated α-tubulin	Sigma-Aldrich	Cat. #: T7451; RRID:AB_609894	IF (1:2000)
Antibody	rabbit polyclonal anti-SFTPC	Merck-Millipore	Cat. #: AB3786; RRID:AB_91588	IF (1:500)
Antibody	mouse monoclonal anti-KRT5 MA5-17057,	Thermo Fisher Scientific	Cat. #: MA5-17057; RRID:AB_2538529	IF (1:200)
Antibody	mouse monoclonal anti-CD45 FITC conjugated	eBioscience	Cat. #: 11-0451-85; RRID:AB_465051	FC (0,05 μg)
Antibody	mouse monoclonal anti-CD11b PE conjugated	eBioscience	Cat. #: 12-0112-82; RRID:AB_2734869	FC (0,05 μg)
Antibody	donkey polyclonal anti-rabbit Alexa Fluor 488	Molecular Probes	Cat. #: A21206; RRID:AB_141708	IF (1:500)
Antibody	donkey polyclonal anti-goat Alexa Fluor 568	Molecular Probes	Cat. #: A11057; RRID:AB_142581	IF (1:500)
Antibody	donkey polyclonal anti-rabbit Alexa Fluor 647	Molecular Probes	Cat. #: A31573; RRID:AB_2536183	IF (1:500)
Antibody	donkey polyclonal anti-mouse Alexa Fluor 647	Molecular Probes	Cat. #: A31571; RRID:AB_162542	IF (1:500)
Antibody	donkey polyclonal anti-mouse Alexa Fluor 568	Abcam	Cat. #: ab175700	IF (1:500)
Sequence-based reagent	Digital droplet PCR primers	This paper	Kras^Q61R^ mutation detection	Forward: ATCTGACGTGCTTTGCCTGT, Reverse: CCCTCCCCAGTTCTCATGTA
Sequence-based reagent	Digital droplet PCR probe	This paper	Kras^Q61R^mutation detection	sequence: GACACAGCAGGTCAAGAGGAGTACA
Sequence-based reagent	Digital droplet PCR primers and probe	Bio-Rad Laboratories	Registration #: dCNS685684912	Tomato allele detection
Sequence-based reagent	Quantitative PCR	This paper	*Scgb1a1* gene	Forward: ATCACTGTGGTCATGCTGTCC, Reverse: GCTTCAGGGATGCCACATAAC
Sequence-based reagent	Quantitative PCR	This paper	*Sftpc* gene	Forward: TCGTTGTCGTGGTGATTGTAG, Reverse: TCGTTGTCGTGGTGATTGTAG
Sequence-based reagent	Quantitative PCR	This paper	*Gusb* gene	Forward: TTACTTTAAGACGCTGATCACC, Reverse: ACCTCCAAATGCCCATAGTC
Commercial assay or kit	GenElute Mammalian Genomic DNA Minipreps Kit	Sigma-Aldrich	Cat. #: G1N70	
Commercial assay or kit	RNeasy Mini Kit	Qiagen	Cat. #: 74106	
Commercial assay or kit	SYBR FAST qPCR Kit	Kapa Biosystems	Cat. #: KK4600	
Commercial assay or kit	MycoAlert Mycoplasma Detection Kit	LONZA	Cat. #: LT07-318	
Chemical compound, drug	Urethane, ethyl carbamate (EC)	Sigma-Aldrich	Cat. #: U2500	1 g/Kg
Chemical compound, drug	3-methylcholanthrene (MCA)	Sigma-Aldrich	Cat. #: 442388	15 mg/Kg
Chemical compound, drug	Butylated hydroxytoluene (BHT)	Sigma-Aldrich	Cat. #: W218405	200 mg/Kg
Chemical compound, drug	Naphthalene	Sigma-Aldrich	Cat. #: 84679	250 mg/Kg
Chemical compound, drug	Bleomycin A2	Calbiochem	Cat. #: 203401	0.08 units
Software, algorithm	Transcriptome Analysis Console Software	https://www.thermofisher.com/tw/zt/home/life-science/microarray-analysis/microarray-analysis-instruments-software-services/microarray-analysis-software/affymetrix-transcriptome-analysis-console-software.html	RRID:SCR_016519	
Software, algorithm	FlowJo software	TreeStar	RRID:SCR_008520	
Software, algorithm	FloMax Software	Partec	RRID:SCR_014437	
Software, algorithm	Broad Institute pre-ranked GSEA module software	http://software.broadinstitute.org/gsea/index.jsp		Subramanian et al., 2005
Software, algorithm	NRECON software	Bruker		
Software, algorithm	CT analysis (Ctan) software	Bruker		
Software, algorithm	CTVox software	Bruker		
Software, algorithm	QuantaSoft	Bio-Rad Laboratories (http://www.bio-rad.com/en-gr/sku/1864011-quantasoft-software-regulatory-edition?ID=1864011)		
Software, algorithm	G*power	http://www.gpower.hhu.de/	RRID:SCR_013726	Faul et al., 2007
Software, algorithm	GraphPad Prism	http://www.graphpad.com/	RRID:SCR_002798	Version 8
Software, algorithm	Fiji	http://fiji.sc	RRID:SCR_002285	PMID: 22743772
Software, algorithm	Living Image software	Perkin-Elmer (http://www.perkinelmer.com/catalog/category/id/living%20image%20software)	RRID:SCR_014247	Version 4.2
Other	Microarray data	This paper	Gene Expression Omnibus (GEO) accession ID: GSE94981	LUAD cells, bone marrow derived macrophages (BMDM), and tracheal AEC cells
Other	Microarray data	Gene Expression Omnibus (GEO)	Accession ID: GSE82154; GSE55459; GSE46749; GSE18816; GSE43458	*M. musculus* ATII cells; *H. sapiens* AEC cells; *H. sapiens* ATII cells;*H. sapiens*AMΦ; *H. sapiens* non-smokers lung and LUAD
Other	GeneChip Mouse Gene 2.0 ST array; GeneChip Human Gene1.0 ST array	Thermo Fisher Scientific	Cat. #: 902119; Cat. #: 901085	
Other	Hoechst33258 nuclear dye	Sigma-Aldrich	Cat. #: 14530	1:5000
Other	D-Luciferin potassium salt	Gold Biotechnology	Cat. #: LUCK-100	1 mg
Other	Trizol	Thermo Fisher Scientific	Cat. #: 15596026	

### Key resources table

All raw data used to generate the main Figures and Figure Supplements are provided as *.xlsx Source Data files.

### Study approval

All mice were bred at the Center for Animal Models of Disease of the University of Patras. Experiments were designed and approved *a priori* by the Veterinary Administration of the Prefecture of Western Greece (approval numbers 3741/16.11.2010, 60291/3035/19.03.2012, and 118018/578/30.04.2014) and were conducted according to Directive 2010/63/EU (http://eur-lex.europa.eu/legal-content/EN/TXT/?qid=1486710385917&uri=CELEX:32010L0063). Male and female experimental mice were sex-, weight (20–25 g)-, and age (6–12 week)-matched. *n* = 588 experimental and *n* = 165 breeder mice were used for this report. Sample size was calculated using power analysis on G*power. Experiments were randomized across different cages and mouse lungs were always examined by two blinded researchers. Sample numbers are included in the figures and figure legends. Archival tissue samples of patients with LUAD (Giopanou et al., 2015) that underwent surgical resection with curative intent between 2001 and 2008 at the University Hospital of Patras were retrospectively enrolled. The observational protocol for these studies adhered to the Helsinki Declaration and was approved by the Ethics Committee of the University Hospital of Patras, and all patients gave written informed consent.

### Reagents

Urethane, ethyl carbamate, EC, CAS# 51-79-6; 3-methylcholanthrene, 3-methyl-1,2-dyhydrobenzo[j]aceanthrylene, MCA, CAS# 56-49-5; butylated hydroxytoluene, 2,6-Di-tert-butyl-4-methylphenol, BHT, CAS# 128-37-0; naphthalene, CAS# 91-20-3, and Hoechst33258 nuclear dye (CAS# 23491-45-4), were from Sigma-Aldrich (St. Louis, MO). Bleomycin A2, ((3-{[(2'-{(5S,8S,9S,10R,13S)−15-{6-amino-2- [(1S)−3-amino-1-{[(2S)−2,3-diamino-3-oxopropyl]amino}−3-oxopropyl] −5-methylpyrimidin-4-yl}−13-[{[(2R,3S,4S,5S,6S)−3-{[(2R,3S,4S,5R,6R)−4-(carbamoyloxy)−3,5-dihydroxy-6- (hydroxymethyl) tetrahydro-2H-pyran-2-yl]oxy} −4,5-dihydroxy-6-(hydroxymethyl) tetrahydro-2H-pyran-2-yl]oxy} (1H-imidazol-5-yl)methyl]−9-hydroxy-5-[(1R)−1-hydroxyethyl]−8,10-dimethyl-4,7,12,15-tetraoxo-3,6,11,14-tetraazapentadec-1-yl}−2,4'-bi-1,3-thiazol-4-yl)carbonyl]amino}propyl) (dimethyl)sulfonium; CAS #9041-93-4, was from Calbiochem (Darmstadt, Germany). D-Luciferin potassium salt, (4S)−2-(6-hydroxy-1,3-benzothiazol-2-yl)−4,5-dihydrothiazole-4-carboxylic acid, CAS #2591-17-5, was from Gold Biotechnology (St. Louis, MO).

### Experimental mice

C57BL/6J (C57BL/6; #000664), FVB/NJ (FVB; #001800), *B6.129(Cg)-Gt(ROSA)26Sor^tm4(ACTB-tdTomato,-EGFP)Luo^/J* [mT/mG; TOMATO; #007676; (Muzumdar et al., 2007)], *FVB.129S6(B6)-Gt(ROSA)26Sor^tm1(Luc)Kael^/J* [LUC; #005125; (Safran et al., 2003)], *B6.129P2-Gt(ROSA)26Sor^tm1(DTA)Lky^/J* [DTA; #009669; (Voehringer et al., 2008)], *B6.129P2-Lyz2^tm1(cre)Ifo^/J* [LYZ2.CRE; #004781; (Desai et al., 2014)], *B6.Cg-Tg(Sox2-cre)1Amc/J* [SOX2.CRE; #008454; (Hayashi et al., 2002)], *B6.Cg-Tg(Vav1-icre)A2Kio/J* [VAV.CRE; #008610; (Ogilvy et al., 1998)], and *B6.Cg-Tg(Nes-cre)1Kln/J* [NES.CRE; #003771; (Tronche et al., 1999)] mice were from Jackson Laboratories (Bar Harbor, MN). *B6;CBA-Tg(Scgb1a1-cre)1Vart/Flmg* (CCSP.CRE; European Mouse Mutant Archive #EM:04965) mice are described elsewhere (Oikonomou et al., 2012) and *Tg(Sftpc-cre)1Blh* (SFTPC.CRE; Mouse Genome Informatics #MGI:3574949) mice were donated by their founder (Okubo et al., 2005). Mice were bred >F12 to the FVB background at the University of Patras Center for Animal Models of Disease.

### Mouse models of lung adenocarcinoma

Six-week-old mice on the C57BL/6 background received ten consecutive weekly intraperitoneal urethane injections (1 g/Kg in 100 μL saline) and were sacrificed 6–7 months after the first injection, or four consecutive weekly intraperitoneal MCA (15 mg/Kg in 100 μL saline) followed by eight consecutive weekly intraperitoneal BHT injections (200 mg/Kg in 100 μL corn oil) and were sacrificed 6–7 months after the first injection. Six-week-old mice on the FVB background received one intraperitoneal urethane injection (1 g/Kg in 100 μL saline) and were sacrificed 6–7 months later (Westcott et al., 2015; Miller et al., 2003; Malkinson et al., 1997; Stathopoulos et al., 2007; Vreka et al., 2018).

### Mouse models of lung injury

Six-week-old mice (C57BL/6 background) received intratracheal bleomycin A2 (0.08 units in 50 μL saline) or intraperitoneal naphthalene (250 mg/Kg in 100 μL corn oil) (Lawson et al., 2005; Rawlins et al., 2009). In addition, preterm mothers of the C57BL/6 background and their offspring were exposed to room air (21% oxygen; control) or 98% oxygen for two days before and four days after birth (Rawlins et al., 2009; Yee et al., 2009). Oxygen levels were continuously monitored. The gas stream was humidified to 40–70% by a deionized water-jacketed Nafion membrane tubing and delivered through a 0.22 μm filter before passage into a sealed Lexan polycarbonate chamber measuring 40 × 25×25 cm and accommodating 25 L gas at a flow rate of 5 L/min, resulting in complete gas exchange every 5 min. Mothers were cycled between litters on 21% and 98% oxygen every 24 hr to prevent oxygen toxicity and to control for nutritional support of the pups. After perinatal hyperoxia, mice remained at room air till sacrificed at eight weeks of age.

### Urethane-induced lung adenocarcinoma cell lines

Lung tumors were dissected from surrounding healthy lung parenchyma under sterile conditions, minced into 1 mm pieces, and cultured at 37°C in 5% CO_2_–95% air using Dulbecco’s Modified Eagle Medium (DMEM), 10% FBS, 2 mM L-glutamine, 1 mM pyruvate, 100 U/mL penicillin, and 100 U/mL streptomycin. All cell lines were immortal and indefinitely phenotypically stable over >18 months and/or 60 passages, and were tumorigenic and metastatic in C57BL/6 mice (Kanellakis et al., 2019). Cell lines were cultured in DMEM supplemented with 10% FBS and 100 IU/mL penicillin/streptomycin and were maintained in humidified incubators at 37°C with 95% air–5% CO_2_. Cell lines were authenticated annually using the short tandem repeat method and were tested negative for *Mycoplasma Spp.* biannually by MycoAlert Mycoplasma Detection Kit (LONZA; Verviers, Belgium).

### Human lung adenocarcinomas

Ten archival formalin-fixed, paraffin-embedded tissue samples of patients with LUAD that underwent surgical resection with curative intent between 2001 and 2008 at the University Hospital of Patras were retrospectively enrolled (Giopanou et al., 2015). The observational protocol for these studies adhered to the Helsinki Declaration and was approved by the Ethics Committee of the University Hospital of Patras, and all patients gave written informed consent.

### Micro-computed tomography

Urethane or saline treated FVB mice were sacrificed six months post urethane/saline injection. Lungs were inflated and fixed with 10% neutral buffered formalin overnight. They were then dehydrated and chemically dried for μCT scanning using a method kindly provided by Jeroen Hostens (Bruker; Kontich, Belgium). Briefly, a gradient ethanol dehydration protocol (from 70–100%) was applied, followed by 2 hr incubation in Hexamethyldisilazane (HMDS; Sigma, St. Louis, MO) and 2 hr air-drying. The dehydrated lungs were then scanned in a Bruker SkyScan 1172 scanner at 41kV without filtration and with 5.94 μm voxel resolution (exposure: 440 ms). The X-ray projections were obtained at 0.35° intervals with a scanning angular rotation of 180° and two frames were averaged for each rotation under a mean of 10 frames per random movement. 3D reconstructions were performed using NRECON software (Bruker). Regions of interest for the whole lung and peripheral lung tissue were defined in the CT analysis software (CTan; Bruker), thresholds applied to detect tissue from background, and a 3D volume rendering of the lungs were performed using the CTVox software (Bruker).

### Structural assessments in murine lungs

Mouse lungs were recoded (blinded) by laboratory members not participating in these studies and were always examined by two independent blinded participants of this study. The results obtained by each investigator were compared, and lungs were re-evaluated if deviant by >20%. Lungs and lung tumors were initially inspected macroscopically under a Stemi DV4 stereoscope equipped with a micrometric scale incorporated into one eyepiece and an AxiocamERc 5 s camera (Zeiss, Jena, Germany) in trans-illumination mode, allowing for visualization of both superficial and deeply-located lung tumors (Stathopoulos et al., 2007; Vreka et al., 2018). Tumor location was charted and diameter (δ) was measured. Tumor number (multiplicity) per mouse was counted and mean tumor diameter per mouse was calculated as the average of individual diameters of all tumors found in a given mouse lung. Individual tumor volume was calculated as πδ^3^/6. Mean tumor volume per mouse was calculated as the average of individual volumes of all tumors found in a given mouse lung, and total lung tumor burden per mouse as their sum. Following macroscopic mapping of lung and lung tumor morphology, lungs of fluorescent reporter mice were imaged on a Leica MZ16F fluorescent stereomicroscope equipped with GFP and RFP filters and a DFC 300FX camera (Leica Microsystems, Heidelberg, Germany) in order to determine their macroscopic fluorescent pattern. Lung volume was measured by saline immersion, and lungs were embedded in paraffin, randomly sampled by cutting 5 μm-thick lung sections (*n* = 10/lung), mounted on glass slides, and stained with hematoxylin and eosin for morphometry and histologic typing of lung tumors. For this, a digital grid of 100 intersections of vertical lines (points) was superimposed on multiple digital images of all lung sections from lung tissue of a given mouse using Fiji academic freeware (https://fiji.sc/). Total lung tumor burden was determined by point counting of the ratio of the area occupied by neoplastic lesions versus total lung area and by extrapolating the average ratio per mouse to total lung volume (Hsia et al., 2010). The results of this stereologic approach were compared with the macroscopic method, and were scrutinized if deviant by >20%. To evaluate alveolar structure and size, we calculated mean linear intercept using randomly sampled hematoxylin and eosin-stained lung sections, as described elsewhere (Hsia et al., 2010). For this, a digital grid of twenty random horizontal lines was superimposed on multiple digital images of all lung sections from lung tissue of a given mouse using Fiji. Mean linear intercept was calculated by counting the intercepts of interalveolar septae with the lines and the formula: Σ{2 x (length of line/number of intercepts)}/total number of lines. All quantifications were done by counting at least five random non-overlapping fields of view of at least ten sections per lung.

### Histology and molecular phenotyping

For histology, lungs were inflated to 20 cmH_2_O pressure that provides for a lung volume equivalent to the resting volume of the lungs (a.k.a. functional residual capacity in humans) and enables precise histologic observations on airway and alveolar structure avoiding false interpretations resulting from the study of compressed or over-inflated lungs (Hsia et al., 2010). Subsequently, lungs were fixed with 10% formalin overnight and were embedded in paraffin. Five-μm-thick paraffin sections were then counterstained with hematoxylin and eosin (Sigma, St. Louis, MO) and mounted with Entellan New (Merck Millipore, Darmstadt, Germany). For immunofluorescence, lungs were inflated with a 2:1 mixture of 4% paraformaldehyde:Tissue-Tek (Sakura, Tokyo, Japan), fixed in 4% paraformaldehyde overnight at 4°C, cryoprotected with 30% sucrose, embedded in Tissue-Tek and stored at −80°C. Ten-μm cryosections were then post-fixed in 4% paraformaldehyde for 10 min, treated with 0.3% Triton X‐100 for 5 min, and incubated in blocking solution containing 10% fetal bovine serum (FBS), 3% bovine serum albumin (BSA), 0.1% polyoxyethylene (20) sorbitanmonolaurate (Tween 20) in 1x phosphate-buffered saline (PBS) for 1 hr. Following labeling with the indicated primary antibodies overnight at 4°C, sections were incubated with fluorescent secondary antibodies, counterstained with Hoechst 33258 and mounted with Mowiol 4–88 (Calbiochem, Darmstadt, Germany). The following primary antibodies were used: rabbit anti-proliferating cell nuclear antigen (PCNA, 1:3000 dilution, ab2426, Abcam, London, UK), rabbit anti-LYZ2 (1:50 dilution, ab108508, Abcam), rabbit anti-KRT5 (1:200 dilution, ab53121, Abcam), rabbit anti-SFTPC (1:200 dilution, sc-13979, Santa Cruz, Dallas, TX), rabbit anti-CCSP (1:200 dilution, sc-25555, Santa Cruz), goat anti-CCSP (1:1000 dilution, sc-9772, Santa Cruz), mouse anti-acetylated α-tubulin (1:2000 dilution, T7451, Sigma-Aldrich, St. Lewis, MO), rabbit anti-SFTPC (1:500 dilution, AB3786, Merck-Millipore, Burlington, MA), and mouse anti-KRT5 (1:200 dilution, MA5-17057, Thermo Fisher Scientific, Waltham, MA). Alexa Fluor donkey anti-rabbit 488 (A21206, Thermo Fisher Scientific), Alexa Fluor donkey anti-mouse 568 (ab175700, Abcam), Alexa Fluor donkey anti-goat 568 (A11057, Thermo Fisher Scientific), Alexa Fluor donkey anti-rabbit 647 (A31573, Thermo Fisher Scientific), and Alexa Fluor donkey anti-mouse 647 (A31571, Thermo Fisher Scientific) secondary antibodies were used at 1:500 dilution. For isotype control, the primary antibody was omitted. Bright-field images were captured with an AxioLab.A1 microscope connected to an AxioCamERc 5 s camera (Zeiss, Jena, Germany) whereas fluorescent microscopy was carried out either on an Axio Observer D1 inverted fluorescent microscope (Zeiss, Jena, Germany) or a TCS SP5 confocal microscope (Leica Microsystems, Wetzlar, Germany) with 20x, 40x and 63x lenses. Digital images were processed with Fiji. All quantifications of cellular populations were obtained by counting at least five random non-overlapping bronchial-, alveolar-, hyperplasia-, or tumor- containing fields of view per section.

### Pulmonary function testing

Following anesthesia induced by intraperitoneal ketamine (100 mg/Kg) and xylazine (10 mL/Kg) and tracheostomy, mice were mechanically ventilated by a Flexivent rodent ventilator (Scireq, Montreal, Ontario, Canada). The whole procedure, described elsewhere (Manali et al., 2011), lasted 15 min. After a 3 min run-in period of ventilation with 21% oxygen, a tidal volume of 10 mL/Kg, a respiratory rate of 150 breaths/min, and a positive end-expiratory pressure of 3 cmH_2_O, paralysis was induced using 8 mg/Kg intraperitoneal succinyl choline, and total respiratory system impedance was obtained by applying an 8-sec-long pseudorandom frequency oscillation (0.5–19.75 Hz) to the airway opening. Thirty seconds prior to initiation of measurements, lung volume history was once controlled by a 6-sec-long inflation to 30 cm H_2_O pressure. Measurements were repeated thrice at 60 s intervals and were averaged. Data were fit into the constant phase model in order to fractionate total respiratory input impedance into airways resistance (Raw) and tissue damping and elastance coefficients. To obtain pressure-volume (PV) curves, the respiratory system was incrementally inflated and deflated to 40 mL/Kg total volume at seven steps each and airway pressures were recorded on each volume change. The slope of the linear portion of expiratory PV curves, which represents static compliance (Cst), a measure of airspace function, was calculated manually. Operators were blinded to animal genotype.

### Digital droplet (dd)PCR

TOMATO, GFP;CCSP.CRE, and GFP;LYZ2.CRE mice (FVB strain) received one intraperitoneal injection of urethane (1 g/Kg) and lungs were then harvested one and two weeks post-urethane, homogenized, and subjected to DNA extraction and purification using GenElute Mammalian Genomic DNA Miniprep Kit (Sigma-Aldrich, St. Louis, MO). DNA concentration and quality were assessed using a Nanodrop 1000 spectrophotometer (Thermo Fisher Scientific,Waltham, MA). DNA concentration was converted to number of diploid copies according to the formula: DNA (ng/µL)/weight of mouse diploid genome (3.9 pg). Digital droplet PCR protocol and analysis was performed as described previously using reagents, equipment and software from BioRad Laboratories Inc (Hercules, CA) (Mazaika and Homsy, 2014). In brief, 20000 genome copies were used. Samples were normalized internally according to the number of accepted droplets and inter-sample normalization was performed according to the formula [x-min(x)]/[max(x)-min(x)],where x represents the actual, min(x) the minimum, and max(x) the maximum number of accepted droplets. The data were reported as % positive/accepted droplets. Sequences of *Kras*^Q61R^ primers and probe were: *Kras*^Q61R^ forward: ATCTGACGTGCTTTGCCTGT, *Kras*^Q61R^ reverse: CCCTCCCCAGTTCTCATGTA, and *Kras*^Q61R^ probe: GACACAGCAGGTCAAGAGGAGTACA. The *Rosa^mT^* assay is registered as dCNS685684912 (Bio-Rad) with MIQE context: seq1:195–315:+CCAGTTCATGTACGGCTCCAAGGCGTACGTGAAGCACCCCGCCGACATCCCCGATTACAAGAAGCTGTCCTTCCCCGAGGGCTTCAAGTGGGAGCGCGTGATGAACTTCGAGGACGGCGGTCT. Primers and fluorescently labeled probes were combined in a mixture containing 18 μM forward and reverse primers and 5 μM labeled probes (20x primer/Taqman probe mix). Reactions were assembled to contain 12.5 μL 2x ddPCR mix no-UTP, 1.25 μL 20x *Kras*^Q61R^primer/Taqman probe Mix, 1.25 μL 20x *Rosa^mT^* custom primer/Taqman probe Mix and 10 μL DNA diluted in nuclease-free water. The ddPCR protocol included a first denaturation step at 95°C for 10 min followed by 40 cycles of denaturation at 95°C for 30 s and 40 cycles of annealing at 62.5°C for 60 s, and was performed in a BioRad T100 Thermal cycler. Results were analyzed with a BioRad QX100 droplet reader using the QuantaSoft software. The amplitude gathering thresholds of positive droplets were set at 3500 for the *Rosa^mT^* and at 10000 for the *Kras*^Q61R^ probe, according to the manufacturer’s instructions.

### Bronchoalveolar lavage (BAL)

BAL was performed using three sequential aliquots of 1000 µL sterile ice-cold phosphate-buffered saline (PBS). Fluid was combined and centrifuged at 260 g for 10 min to separate cells from supernatant. The cell pellet was resuspended in 1 ml PBS containing 2% fetal bovine serum, and the total cell count was determined using a grid hemocytometer according to the Neubauer method. Cell differentials were obtained by counting 400 cells on May-Grünwald-Giemsa-stained cytocentrifugal specimens. Total BAL cell numbers were calculated by multiplying the percentage of each cell type by total BAL cell number (Stathopoulos et al., 2007; Vreka et al., 2018).

### Bioluminescence imaging

LUC;CCSP.CRE mice, bioluminescent reporters of CCSP-labeled cell mass, received one intraperitoneal injection of saline (100 μL saline) or urethane (1 g/Kg in 100 μL saline) and were serially imaged before treatment start, and at 150 and 210 days into treatment. Imaging was done on a Xenogen Lumina II (Perkin-Elmer, Waltham, MA) 5–20 min after delivery of 1 mg D-Luciferin sodium in 100 μL of sterile water to the retro-orbital vein, and data were analyzed using Living Image v.4.2 (Perkin-Elmer, Waltham, MA) (Stathopoulos et al., 2007; Vreka et al., 2018).

### qPCR and microarrays

Triplicate cultures of 10^6^ LUAD cells, BMDM (obtained by 1 week bone marrow incubation with 100 ng/mL M-CSF), and tracheal AEC (obtained by 1 week incubation of stripped mouse tracheal epithelium in DMEM) were subjected to RNA extraction using Trizol (Thermo Fisher) followed by column purification and DNA removal (Qiagen, Hilden, Germany). Whole lungs were homogenized in Trizol followed by the same procedure. Pooled RNA (5 μg) was quality tested (ABI 2000 Bioanalyzer; Agilent Technologies, Sta. Clara, CA), labeled, and hybridized to GeneChip Mouse Gene 2.0 ST arrays (Affymetrix, Sta. Clara, CA). All data were deposited at GEO (http://www.ncbi.nlm.nih.gov/geo/; Accession ID: GSE94981) and were analyzed on the Affymetrix Expression and Transcriptome Analysis Consoles together with previously reported (Frank et al., 2016; Kabbout et al., 2013; Clark et al., 2015; Dancer et al., 2015; Lee et al., 2009) murine ATII and human AEC, ATII, AMΦ, non-smokers lung, and LUAD microarray data (Accession IDs: GSE82154, GSE55459, GSE46749, GSE18816, GSE43458). qPCR was performed using first strand synthesis with specific primers (*Scgb1a1*: ATCACTGTGGTCATGCTGTCC and GCTTCAGGGATGCCACATAAC; *Sftpc*: TCGTTGTCGTGGTGATTGTAG and AGGTAGCGATGGTGTCTGCT; *Gusb*: TTACTTTAAGACGCTGATCACC and ACCTCCAAATGCCCATAGTC) and SYBR FAST qPCR Kit (Kapa Biosystems, Wilmington, MA) in a StepOne cycler (Applied Biosystems, Carlsbad, CA). Ct values from triplicate reactions were analyzed with the 2^-ΔCT^ method relative to *Gusb*.

### Flow cytometry

BAL cells were suspended in 50 μL PBS with 2% FBS and 0.1% NaN_3_, were stained with anti-CD45 (#11-0451-85; eBioscience; Santa Clara, CA) and anti-CD11b (#12-0112-82; eBioscience; Santa Clara, CA) primary antibodies for 20 min in the dark at 0.5 μL antibody per million cells, and were analyzed on a CyFlowML cytometer with a sorter module using FloMax Software (Partec, Darmstadt, Germany) or FlowJo software (TreeStar, Ashland, OR), as described previously (Kanellakis et al., 2019). Perfused lungs were digested in RPMI-1640 medium containing collagenase XI (0.7 mg/mL; Sigma, St. Louis, MO) and type IV bovine pancreatic DNase (30 μg/mL; Sigma, St. Louis, MO) to obtain single-cell suspensions. After treatment with red blood cell lysis buffer (BioLegend; San Diego, CA), single-cell suspensions were analyzed on a LSR II flow cytometer (BD Bioscience, San Diego, CA), and data were examined with FlowJo. Dead cells were excluded using 4,6-diamidino-2-phenylindole (DAPI; Sigma, St. Louis, MO).

### Microarray and gene set enrichment analyses (GSEA)

GSEA was performed with the Broad Institute pre-ranked GSEA module software (http://software.broadinstitute.org/gsea/index.jsp) (Subramanian et al., 2005). In detail, genes significantly expressed (log2 normalized expression >8) in murine tracheal airway cells, ATII cells (Frank et al., 2016), and BMDM were cross-examined against the murine lung and chemical-induced LUAD cell line transcriptomes. In addition, previously reported human AEC, ATII, and AMΦ cellular signatures (Clark et al., 2015; Dancer et al., 2015; Lee et al., 2009) were cross-examined against the previously described transcriptomes of human normal lung tissue from never-smokers and of LUAD (Kabbout et al., 2013).

### Statistical analysis

Sample size was calculated using power analysis on G*power (http://www.gpower.hhu.de/), assuming *α* = 0.05, *β* = 0.05, and effect size *d* = 1.5 (Faul et al., 2007). No data were excluded from analyses. Animals were allocated to treatments by alternation and transgenic animals were enrolled case-control-wise. Data were collected by at least two blinded investigators from samples coded by non-blinded investigators. All data were normally distributed by Kolmogorov-Smirnov test, are given as mean ± SD, and sample size (*n*) always refers to biological and not technical replicates. Differences in frequency were examined by Fischer’s exact and χ^2^ tests and in means by t-test or one-way ANOVA with Bonferroni post-tests. Changes over time and interaction between two variables were examined by two-way ANOVA with Bonferroni post-tests. All probability (*P*) values are two-tailed and were considered significant when p<0.05. All analyses and plots were done on Prism v8.0 (GraphPad, La Jolla, CA).

### Data availability

All raw data produced in this study are provided as *.xlsx source data supplements. The microarray data produced by this study were deposited at GEO (http://www.ncbi.nlm.nih.gov/geo/; Accession ID: GSE94981). Previously reported murine ATII and human AEC, ATII, AMΦ, non-smokers lung, and LUAD microarray data are available at GEO using Accession IDs GSE82154, GSE55459, GSE46749, GSE18816, and GSE43458).

## Data Availability

All raw data produced in this study are provided as *.xlsx source data Supplements. The microarray data produced by this study were deposited at GEO (http://www.ncbi.nlm.nih.gov/geo/; Accession ID: GSE94981). Previously reported (Frank et al., 2016; Clark et al., 2015; Dancer et al., 2015; Lee et al., 2009; Kabbout et al., 2013) murine ATII and human AEC, ATII, AMΦ, non-smokers lung, and LUAD microarray data are available at GEO using Accession IDs GSE82154, GSE55459, GSE46749, GSE18816, and GSE43458). The following dataset was generated: StathopoulosGT2017Epithelial signatures of chemical-induced lung adenocarcinomaGene Expression OmnibusGSE94981 The following previously published datasets were used: FrankDBPengTZeppJASnitowMVincentTLPenkalaIJCuiZHerrigesMJMorleyMPZhouSLuMMMorriseyEE2016Emergence of a Wave of Wnt Signaling that Regulates Lung Alveologenesis by Controlling Epithelial Self-Renewal and Differentiation.NCBI Gene Expression OmnibusGSE8215410.1016/j.celrep.2016.11.001PMC521498227880906 ClarkJGKimKHBasomRSGharibSA2015Plasticity of airway epithelial cell transcriptome in response to flagellin.NCBI Gene Expression OmnibusGSE5545910.1371/journal.pone.0115486PMC432334125668187 DancerRCParekhDLaxSD'SouzaVZhengSBassfordCRParkDBartisDGMahidaRTurnerAMSapeyEWeiWNaiduBStewartPMFraserWDChristopherKBCooperMSGaoFSansomDMMartineauARPerkinsGDThickettDR2015Vitamin D deficiency contributes directly to the acute respiratory distress syndrome (ARDS).NCBI Gene Expression OmnibusGSE4674910.1136/thoraxjnl-2014-206680PMC448404425903964 LeeSMGardyJLCheungCYCheungTKHuiKPIpNYGuanYHancockREPeirisJS2009Systems-level comparison of host-responses elicited by avian H5N1 and seasonal H1N1 influenza viruses in primary human macrophagesNCBI Gene Expression OmnibusGSE1881610.1371/journal.pone.0008072PMC278821320011590 KabboutMGarciaMMFujimotoJLiuDDWoodsDChowCWMendozaGMominAAJamesBPSolisLBehrensCLeeJJWistubaIIKadaraH2013ETS2 mediated tumor suppressive function and MET oncogene inhibition in human non-small cell lung cancer.NCBI Gene Expression OmnibusGSE4345810.1158/1078-0432.CCR-13-0341PMC384643423659968

## Additional files

Transparent reporting form

## Appendix 1

### Abbreviations list and master legend

10.7554/eLife.45571.073 AEC, airway epithelial cells; AMΦ, alveolar macrophages; ANOVA, analysis of variance; ATII, alveolar type II cells; BAL, bronchoalveolar lavage; BASC, bronchoalveolar stem cells; BHT, butylated hydroxytoluene; BMDM, bone-marrow-derived macrophages; C57BL/6 mice, mouse strain inherently resistant to chemical carcinogens; CCSP, Clara cell secretory protein; CCSP.CRE mice, mouse strain in which CRE expression is driven by the *Scgb1a1* promoter; CRE, causes recombination; ddPCR, digital droplet PCR; DTA mice, genetic suicide mouse strain that expresses Diphtheria toxin upon CRE-mediated recombination; EC, ethyl carbamate, urethane; FOXJ1, forkhead box J1; FVB mice, mouse strain inherently susceptible to chemical carcinogens; GFP, green fluorescent protein; GSEA, gene set enrichment analysis; *KRAS*, Kirsten rat sarcoma viral oncogene homologue; KRT5, keratin 5; LUAD, Lung adenocarcinoma; LYZ2, lysozyme 2; LYZ2.CRE mice, mouse strain in which CRE expression is driven by the *Lyz2* promoter; MCA, 3-methylcholanthrene; μCT, micro-computed tomography; *n*, sample size; NES.CRE mice, mouse strain in which CRE expression is driven by the *Nestin* neural promoter; *P*, probability; PCNA, proliferating cell nuclear antigen; LUC mice, mouse strain that reports for CRE-mediated recombination via firefly (Photinus pyralis) luciferase expression; SD, standard deviation; SFTPC, surfactant protein C; SFTPC.CRE mice, mouse strain in which CRE expression is driven by the *Sftpc* promoter; SOX2.CRE mice, mouse strain in which CRE expression is driven by the *Sox2* promoter; TOMATO, red fluorescent TdTomato fluorophore; TOMATO (mT/mG) mice, mouse strain that reports for CRE-mediated recombination via a switch from TOMATO to GFP fluorophore expression; TUBA1A, acetylated tubulin; VAV.CRE mice, mouse strain in which CRE expression is driven by the *Vav1* panhematopoietic promoter.
