# Supplementary material for: Club cells form lung adenocarcinomas and maintain the alveoli of adult mice
Source: eLife. 2019 May 29;8:e45571. doi: 10.7554/eLife.45571 (PMC6606035; doi:10.7554/eLife.45571)
Supplement: Peer review history

# Decision letter

**DOI:** 10.7554/eLife.45571.087

**Affiliations:** King's College LondonUnited Kingdom; UC DenverUnited States

In the interests of transparency, eLife includes the editorial decision letter and accompanying author responses. A lightly edited version of the letter sent to the authors after peer review is shown, indicating the most substantive concerns; minor comments are not usually included.

Thank you for submitting your article "Dual airway and alveolar contributions to adult lung homeostasis and carcinogenesis" for consideration by *eLife*. Your article has been reviewed by three peer reviewers and the evaluation has been overseen by a Reviewing Editor and Jeffrey Settleman as the Senior Editor. The following individual involved in the review of your submission has agreed to reveal their identity: York Miller (Reviewer #3).

The reviewers have discussed the reviews with one another and the Reviewing Editor has drafted this decision to help you prepare a revised submission. While the reviews are generally positive about the findings, all reviewers are united on the view that the manuscript requires a substantial rewrite, as it was incredibly difficult to understand, full of jargon, and is written for an extremely specialist audience, rather than the general readership at *eLife*. Therefore, we offer you the opportunity to rewrite the manuscript in a more general way, with good descriptors of each cell type and labels for them, rather than specific markers. Compared to the main text having too many specific markers, the title is too general and does not state clearly what the novelty of the findings are. We will consider the revised manuscript for publication only if we feel that you can communicate it to a wider audience.

For assistance, York has kindly offered a brief descriptor of what he believes the novelty of the findings are and ways that the text could be improved:

1) They show that mouse lung adenocarcinomas do largely arise from cells expressing a Club cell marker, not those derived from ATII cells. This is done by the lineage tracing, by morphometry and imaging of early tumors and by ablation using targeted expression of diphtheria toxin. These extend and modify previous studies by Carla Kim using IHC and ISH. The importance of this is that if we are going to target early premalignancy, strategies focused on Club cells seem more promising than those targeting ATII cells.

2) Most interesting was the finding that both ATII and Club type cells carry KRAS mutations shortly after urethane carcinogenesis, but that the mutated ATII cells become extinct while the Club cells persist and evolve into adenocarcinomas.

3) There is some transcriptomic evidence to suggest that a similar situation exists in human lung adenocarcinoma. Clearly this is not as rigorous as can be demonstrated in the mouse.

There are several ways this can be greatly improved.

1) Define abbreviations early in manuscript and provide some guidance as to what the import of these markers is.

2) Figures are very small and hard to follow. Unfortunately, this is common across a lot of current literature as authors try to jam a large amount of experimental work into a small space.

3) Some of the figures, such as the one showing the KRAS mutation in ATII and Club cells, could be simplified. The time course graph here is most important.

4) Provide a better introduction to the abbreviations and markers for readers not intimately knowledgeable about this field.

These are a summary of the main points that we will need for you to revise. I include the full reviews of the reviewers, which may also help you to rewrite the paper for your reference alone.

*Reviewer #1:*

The manuscript, "Dual airway and alveolar contributions to adult lung homeostasis and carcinogenesis" by Spella et al., employ specific cell labeling and transcriptomics to identify airway cell types in developing versus wounded versus carcinogen-induced adenocarcinoma induction. Overall, they find that 'airway cells' are required for alveolar maintenance and lung carcinoma development. While the results documented in this manuscript could be compelling and many of the questions that they are asking are fundamental questions in biology and cancer, the written paper and figures were so hard to read that I cannot interpret them. There are no clues to all the acronyms and markers the authors use throughout the paper until potentially the summary at the last figure. It is not the job of the reader to do all the background reading to try to understand what the authors may or may not be trying to say. Therefore, while their findings may be compelling, the authors must find a way to relay a coherent story that is understandable to the wide audience at *eLife*.

Additionally, I have a few comments, based on what I think I may have interpreted correctly.

1) Is it really interesting to find that, "Collectively, these results indicate that airway cells and/or transcriptomic signatures are essential for alveolar maintenance and LUAD development."? Are cells not within the airway expected to contribute to airway development and cancer. Again, this may lie within code words used between people in the lab that they haven't explained to other people. To me, airway cells include all cells within the airway, including stroma.

2) I think that the findings that carcinogenesis could come from faulty repopulation of adult cells following the continuous wounding of airway epithelia to be the most interesting finding of this study, yet it is not tackled directly. Is this something that the authors are saying?

3) The main problem with the paper is that the authors are using drivers of cell lines that they never describe or confirm with markers that are clearly labeled and cited. The reader has no idea what cells they are labeling with what driver and how we know that the labels are acceptable. Without this, the whole study could be a house of cards with interpretations depending on whatever they have decided to call cells.

4) They need to find a way to write in a way that captures the reader's attention so that we understand right upfront what the problem is that they are trying to address and how they will approach it. They could put the drivers and markers into a table, once they show clear evidence what cell type they refer to. Then refer only to the cell type in plain English, not a thousand acronyms.

5) There seems to be a very fuzzy plot throughout. They are using cell lines, ablation of certain cells, Cre-labeled cells and then they come up with very fuzzy conclusions. They might do well to reduce what they are looking at and come up with clear questions to the answers that they think that the experiments answer.

*Reviewer #2:*

The authors studied the distribution of airway cells in lung injury and repair including tumorigenesis. They characterized the dynamics of respiratory epithelial cells in the mouse lung during aging and after challenge with toxic and carcinogens. They developed Cre-reporter mice with the tomato (mT) to GFP (mG) fluorescence expression of six different Cre-driver genes, three of which were successful. They demonstrated expression in airway and alveolar cells at baseline and in chemical carcinogen induced lung ADC in FVB mT/mG mice expressing SCGB1a1 or Lyz2. They demonstrated dissemination of an "airway transcriptomic signature" in the alveoli of carcinogen treated mice by virtue of showing Scgbia1 cells to be CCSP+TUBA1A-SFTPC-. They demonstrated dynamic changes in cellular composition presumably induced by the field of cancerization – tobacco. Scag1a1 cells proliferate after injury including bleomycin, hyperoxia and naphthalene. Lastly, they showed using Dta ablation model that both SCGB1a1 and Lyz2 driven cells are key for tumor development. In a series of clever experiments, they studied epithelial cell signatures, urethane induced in cancer cells vs tracheal explants and ATII cells, and BMDM. Lung ADCs lost expression of epithelial markers and gained EGFR, Myc and Krt18 markers. AEC (but not the ATII and AMΦ) signature was significantly enriched in LUAD compared with healthy lung tissue. AEC gene expression signature predominated over ATII and BMDM signatures in LUAD cells. Finally, the results indicate that both airway and alveolar-restricted programs are needed for structural and functional integrity.

Overall the paper is very interesting and loaded with information. The premise of the work is very strong: "ablation of airway cells distorted alveolar structure and function and protected mice from LUAD development."

The authors should be commended for the novelty of the work and for the potentially transformative findings bringing the plasticity of the conducting airway epithelial cells to the forefront of lung homeostasis and tumorigenesis. The paper does nail down in mice what specific cell type(s) in the conducting airway is at stake.

The findings support that airway cells and/or transcriptomic signatures are essential for alveolar maintenance and LUAD development. This statement may be true in the mouse models generated but the evidence that would suggest that is can be true in humans is insufficiently developed.

"The data indicate remarkable plasticity of adult lung epithelial cells and suggest a central role for airway cells and transcriptional signature (s)in lung regeneration and cancer". It remains unclear as to whether these scgb1A1 cells are migrating to the alveolar space or rather expressing CCSP by regenerating alveolar cells. The question is not settled by the results of these experiments, yet it has huge implications. The data seem to further support the concept of trans-differentiation which is not addressed in the paper. Isolation and transcriptional profile at the single cell level may solve the question.

Upon exposure to urethane the Scgb1a1.cre;Dta and lyz2 were equally protected from developing lung ADC? Why would Lyz2 cells be needed for tumorigenesis is not clear.

The paper is not well written overall and was very challenging to read/ review. The paper is structured in such way that we discuss lung tumors than homeostasis and repair and back to lung ADC. It is quite difficult to follow. There are many grammatical errors and too many shortcuts for expressing new concepts. It would benefit from simplification and from focusing the report on the main findings.

*Reviewer #3:*

The authors examine the evolution of chemically induced lung adenocarcinomas in three informative genetically marked mouse strains: Scgb1a1.Cre, Sftpc.Cre and Lyz2.Cre crossed with a reporter construct mediated by a permanent switch from membranous mT to mG. The genetic marking approach yields somewhat discordant results with immunostaining for marker proteins and provides evidence that airway epithelial cells (Scgb1a1 descendant) are critical in both airway and alveolar repair after injury as well as the development of adenocarcinoma precursors. They support this with both imaging and histochemical analyses that demonstrate a majority of adenocarcinomas appear to originate from airways, not alveoli. Additional evidence is added from diphtheria toxin mediated cell line ablation. Furthermore, they trace the timecourse of the urethane induced KRAS mutation in Scgb1a1 and Sftpc derived cells which demonstrates an impressive extinction of the latter and persistence of the former, raising interesting questions as to the selective pressures driving this evolution. Additional experiments describe the role of Scgb1a1 derived cells in aging and injury repair. To the extent possible, the authors support the relevance of their findings to human lung adenocarcinoma through transcriptomic analysis.

As I am not an expert in cell lineage tracing, there may be issues here which I have missed. I believe that the results provide strong support for the importance of airway epithelial cells, rather than alveolar type II cells, in the genesis of many lung adenocarcinomas. Why the KRAS mutation specifically caused by urethane is maintained in airway epithelial derived cells but is not in alveolar type II cells is fascinating. This is highly relevant to prevention and risk determination. I do not suggest any major additional work.

[Editors' note: further revisions were requested prior to acceptance, as described below.]

Thank you for resubmitting your work entitled "The conducting airway epithelium fosters chemical-induced lung adenocarcinoma and alveolar maintenance in adult mice." for further consideration at *eLife*. Your revised article has been favorably evaluated by Jeffrey Settleman (Senior Editor) and a Reviewing Editor.

The manuscript has been improved but there are some remaining issues that need to be addressed before acceptance, as outlined below:

The revised manuscript is much easier to read. My only remaining issue with the revised paper is that the Title and Abstract are still confusing. Title: The conducting airway epithelium (again that doesn't seem very specific) fosters (what does foster mean? usually it means to adopt for a short time or to nurture. It is still a vague word – do you mean it acts like a seed or a soil? This is not a very cogent title and needs help). Additionally, the Abstract's last two sentences are a jumble. Why not break them up into different sentences so that each has a precise meaning. Finally, 'lucrative'? Is that what is being learnt here? They could be lucrative cells? I think that this needs a good rewrite. My sense is that this is an important paper that should be published but it really deserves to be understood by its readers. Therefore, I have asked for a full copyedit and I think it would be good to make sure that you are stating everything you want to say, especially in the Title and the Abstract, which will get the most readership.

# Reply: Author response

**DOI:** 10.7554/eLife.45571.088

The reviewers have discussed the reviews with one another and the Reviewing Editor has drafted this decision to help you prepare a revised submission. While the reviews are generally positive about the findings, all reviewers are united on the view that the manuscript requires a substantial rewrite, as it was incredibly difficult to understand, full of jargon, and is written for an extremely specialist audience, rather than the general readership at eLife. Therefore, we offer you the opportunity to rewrite the manuscript in a more general way, with good descriptors of each cell type and labels for them, rather than specific markers. Compared to the main text having too many specific markers, the title is too general and does not state clearly what the novelty of the findings are. We will consider the revised manuscript for publication only if we feel that you can communicate it to a wider audience.

We are extremely thankful to you both and our reviewers for the time spent in reviewing our paper, the overall positive appraisal of our findings, and the constructive criticism that helped us immensely in improving the manuscript. We are especially thankful to Dr. York Miller, reviewer #3, who provided additional feedback in order to help us re-write the paper. We have undertaken every effort feasible to address the editorial and reviewers’ comments and are hereby submitting an overtly improved version of the paper, which we hope to be acceptable. In more detail, we have now completely re-written the manuscript and re-designed the figures in order to more effectively convey the findings to a broad readership. Violin plots were included in the new figures, which depict the raw data as well as their distribution.

For assistance, York has kindly offered a brief descriptor of what he believes the novelty of the findings are and ways that the text could be improved:

1) They show that mouse lung adenocarcinomas do largely arise from cells expressing a Club cell marker, not those derived from ATII cells. This is done by the lineage tracing, by morphometry and imaging of early tumors and by ablation using targeted expression of diphtheria toxin. These extend and modify previous studies by Carla Kim using IHC and ISH. The importance of this is that if we are going to target early premalignancy, strategies focused on Club cells seem more promising than those targeting ATII cells.

2) Most interesting was the finding that both ATII and Club type cells carry KRAS mutations shortly after urethane carcinogenesis, but that the mutated ATII cells become extinct while the Club cells persist and evolve into adenocarcinomas.

3) There is some transcriptomic evidence to suggest that a similar situation exists in human lung adenocarcinoma. Clearly this is not as rigorous as can be demonstrated in the mouse.

There are several ways this can be greatly improved.

1) Define abbreviations early in manuscript and provide some guidance as to what the import of these markers is.

2) Figures are very small and hard to follows. Unfortunately, this is common across a lot of current literature as authors try to jam a large amount of experimental work into a small space.

3) Some of the figures, such as the one showing the KRAS mutation in ATII and Club cells, could be simplified. The time course graph here is most important.

4) Provide a better introduction to the abbreviations and markers for readers not intimately knowledgeable about this field.

These are a summary of the main points that we will need for you to revise. I include the full reviews of the reviewers, which may also help you to rewrite the paper for your reference alone.

We carefully abided by the editorial instructions and made all changes requested by Dr. Miller, the other reviewers, and the Editors.

Reviewer #1:

The manuscript, "Dual airway and alveolar contributions to adult lung homeostasis and carcinogenesis" by Spella et al., employ specific cell labeling and transcriptomics to identify airway cell types in developing versus wounded versus carcinogen-induced adenocarcinoma induction. Overall, they find that 'airway cells' are required for alveolar maintenance and lung carcinoma development. While the results documented in this manuscript could be compelling and many of the questions that they are asking are fundamental questions in biology and cancer, the written paper and figures were so hard to read that I cannot interpret them. There are no clues to all the acronyms and markers the authors use throughout the paper until potentially the summary at the last figure. It is not the job of the reader to do all the background reading to try to understand what the authors may or may not be trying to say. Therefore, while their findings may be compelling, the authors must find a way to relay a coherent story that is understandable to the wide audience at eLife.

We thank reviewer #1 for the constructive criticism. In this revised form of the paper we tried to make the manuscript more coherent and the figures more comprehensible. Furthermore, we added tables listing and explaining the protein markers that we used in our studies.

Additionally, I have a few comments, based on what I think I may have interpreted correctly.

1) Is it really interesting to find that, "Collectively, these results indicate that airway cells and/or transcriptomic signatures are essential for alveolar maintenance and LUAD development."? Are cells not within the airway expected to contribute to airway development and cancer. Again, this may lie within code words used between people in the lab that they haven't explained to other people. To me, airway cells include all cells within the airway, including stroma.

We thank the reviewer for this comment, which we tried to clarify in the revised manuscript. The novel concept we are proposing is that airway (or airway-reprogrammed) cells cause cancer in the alveoli, which is opposed to the doctrine that alveolar cells cause cancer in the alveoli (adenocarcinomas). Lungs are grossly comprised by two distinct compartments, the airways or bronchi and the alveolar parenchyma, and their homeostasis has been a longstanding issue in lung biology. A number of pulmonary lineage tracing studies utilizing noxious insults showed that each compartment is supposedly maintaining itself, with airway epithelial cells (AEC) sustaining the airways and alveolar type II (ATII) cells reforming the alveolar parenchyma. In lung adenocarcinoma, studies using genetic mouse models of oncogenic KRAS expression in the respiratory epithelium incriminated different cells as progenitors of lung adenocarcinoma, but these models are marginally human-relevant and their results rest on where the transgene is turned on.

2) I think that the findings that carcinogenesis could come from faulty repopulation of adult cells following the continuous wounding of airway epithelia to be the most interesting finding of this study, yet it is not tackled directly. Is this something that the authors are saying?

Like reviewer #1 so astutely deduces, we believe that airway epithelial cells significantly contribute to adenocarcinoma formation, as they are the first cells to be mutated after carcinogenic insults and this can of course affect the correct repopulation of the adult lung. We hope that in the revised version of our manuscript the interpretation of our findings will be clearer.

3) The main problem with the paper is that the authors are using drivers of cell lines that they never describe or confirm with markers that are clearly labeled and cited. The reader has no idea what cells they are labeling with what driver and how we know that the labels are acceptable. Without this, the whole study could be a house of cards with interpretations depending on whatever they have decided to call cells.

We thank reviewer #1 for the constructive criticism. In this revised version of the paper, we describe our mouse models more explicitly. Furthermore, we have added tables listing and explaining the protein markers that we used.

4) They need to find a way to write in a way that captures the reader's attention so that we understand right upfront what the problem is that they are trying to address and how they will approach it. They could put the drivers and markers into a table, once they show clear evidence what cell type they refer to. Then refer only to the cell type in plain English, not a thousand acronyms.

We thank reviewer #1 for the constructive criticism. According to his/her suggestions, we included tables listing the drivers and protein markers that we used. We have simplified our figures to clearly show the relation of each protein marker to the respective cell type. We now refer to our reporters as “GFP;REPORTER.CRE” in order to show which cell type is GFP labeled.

5) There seems to be a very fuzzy plot throughout. They are using cell lines, ablation of certain cells, Cre-labeled cells and then they come up with very fuzzy conclusions. They might do well to reduce what they are looking at and come up with clear questions to the answers that they think that the experiments answer.

We thank reviewer #1 for the constructive criticism. We hope that the revised form of our manuscript will be more comprehensible.

Reviewer #2:

[…] Overall the paper is very interesting and loaded with information. The premise of the work is very strong. "ablation of airway cells distorted alveolar structure and function and protected mice from LUAD development."

The authors should be commended for the novelty of the work and for the potentially transformative findings bringing the plasticity of the conducting airway epithelial cells to the forefront of lung homeostasis and tumorigenesis. The paper does nail down in mice what specific cell type(s) in the conducting airway is at stake.

We thank reviewer #2 for his/her positive comments and the appreciation of our work.

The findings support that airway cells and/or transcriptomic signatures are essential for alveolar maintenance and LUAD development. This statement may be true in the mouse models generated but the evidence that would suggest that is can be true in humans is insufficiently developed.

We thank reviewer #2 for the constructive criticism. Admittedly, our human transcriptomic analysis is not as rigorous as the respective analysis in mice. However, we believe that this can be explained by the early nature of the human surgical specimens that we used compared to our murine cell lines that represent advanced metastatic tumor-initiating cells. We agree with the reviewer’s comment and included the limitations of our human data in the revised Discussion section.

"The data indicate remarkable plasticity of adult lung epithelial cells and suggest a central role for airway cells and transcriptional signature (s)in lung regeneration and cancer". It remains unclear as to whether these scgb1A1 cells are migrating to the alveolar space or rather expressing CCSP by regenerating alveolar cells. The question is not settled by the results of these experiments, yet it has huge implications. The data seem to further support the concept of trans-differentiation which is not addressed in the paper. Isolation and transcriptional profile at the single cell level may solve the question.

We thank reviewer #2 for the constructive criticism and the effort to improve our manuscript. Admittedly, our data set up doesn’t address the issues of CCSP+ cells migration to the alveolar space and trans-differentiation. Single cell transcriptional profiling is one of our future steps, but we could manage to perform such studies to date. We have included this limitation in the revised Discussion section and have clearly stated that we do not definitively address the migration versus CCSP expression by regenerating alveolar cells issue.

Upon exposure to urethane the Scgb1a1.cre;Dta and lyz2 were equally protected from developing lung ADC? Why would Lyz2 cells be needed for tumorigenesis is not clear.

LYZ2-expressing cells in the lungs are alveolar macrophages, but also half of alveolar type II cells (Nature 2014, 507:190-194). LYZ2+ alveolar cells have been previously shown to play a major role in adenocarcinoma formation in studies using mouse models of genetic activation of *KRAS* in alveolar cells. This finding is corroborated in our studies, since we show that a portion of tumors are LYZ2-labeled and LYZ2-ablated mice have less tumors in response to urethane. However, we show that many lung tumors arise independent from LYZ2+ cells, and that LYZ2+ cells in the alveoli have genetic airway labeling. The data collectively support previous findings, but imply that airway cells can be at the base of LYZ2+ cells in the alveoli.

The paper is not well written overall and was very challenging to read/ review. The paper is structured in such way that we discuss lung tumors than homeostasis and repair and back to lung ADC. It is quite difficult to follow. There are many grammatical errors and too many shortcuts for expressing new concepts. It would benefit from simplification and from focusing the report on the main findings.

We thank reviewer #2 for the constructive criticism. We hope that the revised form of our manuscript will be more comprehensible.

Reviewer #3:

[…] As I am not an expert in cell lineage tracing, there may be issues here which I have missed. I believe that the results provide strong support for the importance of airway epithelial cells, rather than alveolar type II cells, in the genesis of many lung adenocarcinomas. Why the KRAS mutation specifically caused by urethane is maintained in airway epithelial derived cells but is not in alveolar type II cells is fascinating. This is highly relevant to prevention and risk determination. I do not suggest any major additional work.

We sincerely thank reviewer #3, Dr. York E. Miller, for these positive comments and the appraisal of our work. This is especially important for us to keep going, since this comment stems from a pioneer in the field.

[Editors' note: further revisions were requested prior to acceptance, as described below.]

The revised manuscript is much easier to read. My only remaining issue with the revised paper is that the Title and Abstract are still confusing. The Title: The conducting airway epithelium (again that doesn't seem very specific) fosters (what does foster mean? usually it means to adopt for a short time or to nurture. It is still a vague word – do you mean it acts like a seed or a soil? This is not a very cogent Title and needs help). Additionally, the Abstract's last two sentences are a jumble. Why not break them up into different sentences so that each has a precise meaning. Finally, 'lucrative'? Is that what is being learnt here? They could be lucrative cells? I think that this needs a good rewrite. My sense is that this is an important paper that should be published but it really deserves to be understood by its readers. Therefore, I have asked for a full copyedit and I think it would be good to make sure that you are stating everything you want to say, especially in the Title and the Abstract, which will get the most readership.

We hereby resubmit a revised version of the above-referenced manuscript. As requested, we completely rewrote the Title and Abstract, and made additional edits throughout the paper. We are extremely thankful to you both for the time spent in reviewing our paper and for your positive appraisal of the work.

We are now clearly saying what we mean and hope the manuscript to be acceptable.
